# Comprehensive Study on the Reinforcement of Electrospun PHB Scaffolds with Composite Magnetic Fe_3_O_4_–rGO Fillers: Structure, Physico-Mechanical Properties, and Piezoelectric Response

**DOI:** 10.1021/acsomega.2c05184

**Published:** 2022-11-04

**Authors:** Artyom S. Pryadko, Yulia R. Mukhortova, Roman V. Chernozem, Lada E. Shlapakova, Dmitry V. Wagner, Konstantin Romanyuk, Evgeny Y. Gerasimov, Andrei Kholkin, Roman A. Surmenev, Maria A. Surmeneva

**Affiliations:** † Physical Materials Science and Composite Materials Center, Research School of Chemistry & Applied Biomedical Sciences, Tomsk Polytechnic University, Tomsk634050, Russia; ‡ Tomsk State University, Tomsk634050, Russia; § School of Natural Sciences and Mathematics, Ural Federal University, Ekaterinburg620000, Russia; ∥ Department of Physics & CICECO−Aveiro Institute of Materials, University of Aveiro, Aveiro3810-193, Portugal; ⊥ Boreskov Institute of Catalysis SB RAS, Novosibirsk630090, Russia; # International Research & Development Center of Piezo- and Magnetoelectric Materials, Research School of Chemistry and Applied Biomedical Sciences, Tomsk Polytechnic University, Tomsk634050, Russia

## Abstract

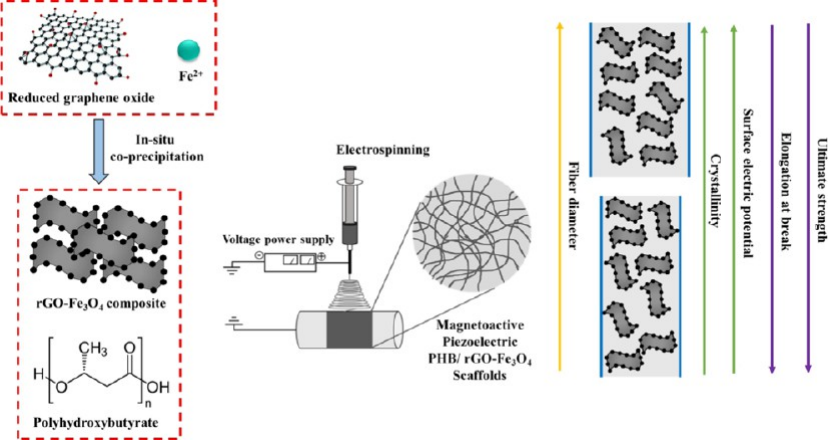

This is a comprehensive study on the reinforcement of electrospun poly(3-hydroxybutyrate) (PHB) scaffolds with a composite filler of magnetite–reduced graphene oxide (Fe_3_O_4_–rGO). The composite filler promoted the increase of average fiber diameters and decrease of the degree of crystallinity of hybrid scaffolds. The decrease in the fiber diameter enhanced the ductility and mechanical strength of scaffolds. The surface electric potential of PHB/Fe_3_O_4_–rGO composite scaffolds significantly increased with increasing fiber diameter owing to a greater number of polar functional groups. The changes in the microfiber diameter did not have any influence on effective piezoresponses of composite scaffolds. The Fe_3_O_4_–rGO filler imparted high saturation magnetization (6.67 ± 0.17 emu/g) to the scaffolds. Thus, magnetic PHB/Fe_3_O_4_–rGO composite scaffolds both preserve magnetic properties and provide a piezoresponse, whereas varying the fiber diameter offers control over ductility and surface electric potential.

## 1 Introduction

A tissue engineering scaffold serves as a substitute of the native extracellular matrix and plays a crucial role in tissue regeneration by providing temporary support for cells during natural extracellular-matrix formation.^1^ For tissue engineering applications, the design of a scaffold should meet some basic requirements, such as biodegradability, biocompatibility, and mechanical properties that match those of a native tissue.^2^

Lately, electrospinning has been receiving much attention because it allows the fabrication of polymeric nano- and microfibrous scaffolds.^3^ Such scaffolds are the most suitable platform for tissue engineering applications owing to their physical, chemical, and mechanical properties making them desirable for cell–cell and cell–matrix interactions.^4^ Electrospun scaffolds have various advantages such as a high surface area-to-volume ratio and a three-dimensional (3D) microenvironment with a controllable and uniform structure meeting the needs of an injury site.^5^ Electrospun scaffolds consist of interconnected fibers that provide a superficial porous structure enabling the transport and exchange of nutrients and growth factors.^6^

Magnetically responsive scaffolds are a class of stimuli-responsive materials for tissue engineering applications and can provide targeted and tailored stimulation of cells and tissues after implantation using an external magnetic field. Varying the external magnetic field parameters gives a researcher precise control over the cellular response. Furthermore, a synergistic combination of magnetic particles and piezoelectric polymers makes it possible to develop materials that allow generating local piezoelectric surface potentials on the scaffolds during magnetic-field exposure in a bioreactor; this approach can be useful for mimicking specific microenvironments and may stimulate the regeneration of specific tissues. It has been shown that Terfenol-d–poly(vinylidene fluoride-*co*-trifluoroethylene) composites can deliver mechanical and electrical stimuli to MC3T3-E1 preosteoblasts and that these stimuli can be triggered remotely by an applied magnetic field; cell proliferation is enhanced up to 25% when cells are cultured under mechanical (up to 110 ppm) and electrical stimulation (up to 0.115 mV).^7^ In another work, during the application of magnetic stimuli, 3D PVDF–CoFe_2_O_4_ scaffolds with different pore sizes promoted the proliferation of preosteoblasts via a local magnetomechanical response of the scaffolds, and this technique induced a proper cellular mechano- and electro-transduction process.^8^

Polyhydroxybutyrate (PHB) is a piezoelectric, thermoplastic, biocompatible, and biodegradable polymer of the polyhydroxyalkanoate family and is produced within the cellular structure of prokaryotes (bacteria).^9^ Biodegradable scaffolds made of PHB can support long-term tissue regeneration owing to their slow degradation rate.^10^ A degradation product of PHB—d-3-hydroxybutyric acid—is a natural constituent of human blood, is nontoxic when present in body fluids, and exerts no inflammatory effects.^11^ Due to its piezoelectric properties, PHB can be deformed in an external electric field or can generate electrical charges upon mechanical stress for the electric stimulation of cells.^12^ The piezoelectric properties of PHB are determined by its asymmetric crystal structure. Piezoelectric properties and surface potentials of piezoelectric electrospun polymer scaffolds depend on the diameter of the fibers and crystallinity of the material in question; for example, poly-l-lactic acid (PLLA) nanotubes with lower crystallinity and smaller diameter have a lower surface potential.^13^ Moreover, the piezoresponse of polymer scaffolds can be enhanced by supplementation with a filler, which affects the polymer structure. For instance, PHB scaffolds functionalized with reduced graphene oxide (rGO) yield a stronger piezoresponse.^14^ Copolymers, such as poly(3-hydroxybutyrate-*co*-3-hydroxyvalerate) (PHBV), also give an enhanced piezoresponse of nanofibrous scaffolds after the addition of graphene oxide (GO) as compared to pure PHBV scaffolds.^15^ Furthermore, structural changes upon the addition of fillers affect the physico-mechanical properties of a polymer. In this regard, a large number of studies have been devoted to determining the influence of fillers, such as GO,^16^ carbon nanotubes (CNTs),^17−19^ magnetite,^20−22^ and hydroxyapatite (HA),^23,24^ on the mechanical properties of composite polymers. Graphene-based materials’ remarkable mechanical characteristics, which are related to the flexible hexagonal network of sp^2^-carbon atoms, make them appealing candidate materials for regenerative medicine.^16,25^ Zine and Sinha have managed to overcome the inherent brittleness of PHBV by the incorporation of GO, which may immobilize the polymer chains and hence improve their flexibility.^16^ The addition of multiwalled CNTs to PHB scaffolds greatly enhances their mechanical strength and elastic modulus.^25^

The crystallinity of polymers is reported to influence their biodegradation rate^26^ and mechanical properties.^27,28^ Generally, in semicrystalline polymers, a higher degree of crystallinity yields a lower content of free volume and therefore an increase in stiffness.^28^ PHB is a semicrystalline thermoplastic polymer, which, in contrast to amorphous polymers, can crystallize from a melt or solution in the form of spherulites, and the crystallization is time-dependent.^29^ Inherent drawbacks of PHB are thermal instability and brittleness. The thermal instability of PHB is attributed to its low degradation temperature, which is very close to its melting temperature.^30^ The brittleness of PHB is ascribed to (i) secondary crystallization during storage; (ii) low nucleation density and consequently large spherulites conducive to inter-spherulitic cracks; and (iii) glass temperature of PHB being close to room temperature.^27,29,30^ Because these drawbacks limit biomedical applications of PHB, it is crucial to understand how various factors, namely, the fiber diameter and filler incorporation, alter the crystallinity of PHB-based scaffolds. To date, a number of researchers have investigated the crystallization of polyhydroxyalkanoates in the presence of various particles;^31−33^ however, the results show some discrepancies. On the one hand, these particles may act as additional nucleating centers for the polymer, thereby promoting its crystallization.^31,34−37^ On the other hand, with increasing filler content, an abrupt reduction in crystallinity occurs due to the emergence of agglomerates, which restrain polymer chains’ mobility and hinder crystallization.^32−36,38,39^ In addition, dimensional constraints are known to limit the crystallization activity of PHB.^40,41^ The fiber size dependence of PLLA crystallinity has been documented,^42^ but the underlying mechanism is still not fully understood.

Magnetite (Fe_3_O_4_) nanoparticles are commonly used as fillers in magnetoresponsive biomaterials owing to their high magnetization, biocompatibility, unique physico-chemical properties, and chemical stability under physiological conditions. Fe_3_O_4_–rGO composites are employed in various biomedical applications. For example, a high Fe_3_O_4_ content is effective in improving the osteoconductivity of a Fe_3_O_4_–rGO nanocomposite.^43^ Much research has been devoted to the use of composites in the areas of drug loading,^44^ separation, and chemical extraction,^30^ sensors, and biosensors because of the synergistic effect of the rGO matrix and Fe_3_O_4_,^45^ which is aimed at enhancing the conductivity and ionic diffusion, thus giving a material with superior electrochemical performance. Accordingly, Fe_3_O_4_–rGO composites have various promising applications due to a synergistic combination of high saturation magnetization of Fe_3_O_4_ with high conductivity and high surface-to-volume ratio of rGO.

Thus, PHB/Fe_3_O_4_–rGO composite scaffolds are promising candidates for magnetoactive biomaterials; however, such biomaterials have not been reported so far. To the best of our knowledge, a comprehensive investigation into the effects of the Fe_3_O_4_–rGO filler and fiber diameter on the morphology, structure, and physico-mechanical, magnetic, and piezoelectric properties of electrospun PHB scaffolds has not been reported so far. Therefore, the present work addresses these effects.

## 2 Materials and Methods

### 2.1 Materials

Iron(III) chloride hexahydrate (FeCl_3_·6H_2_O), iron(II) sulfate heptahydrate (FeSO_4_·7H_2_O), urea, sodium hydroxide, and rGO (BET surface area, 10^3^ m^2^/g; conductivity, 7111 S/m) were purchased from Sigma-Aldrich and used without further purification. Deionized water prepared by means of a Millipore Milli-Q system (Germany) was employed in all experiments.

### 2.2 Fabrication of Fe_3_O_4_–rGO Composites

#### 2.2.1 rGO Surface Treatment before Fe_3_O_4_–rGO Composite Synthesis

The surface of rGO was prepared via the impregnation method 1 day before the synthesis of Fe_3_O_4_–rGO composites. For this purpose, 0.2 g of rGO was treated with 15 mL of 0.25 M NaOH with ultrasonication for 4 h and then heated on a magnetic stirrer for 20 h at 60 °C.

#### 2.2.2 Synthesis of the Fe_3_O_4_–rGO Composite

Magnetite particles were generated directly on the surface of rGO flakes via mixing of a suspension of the treated rGO with solutions of iron salts. To this end, 3.378 g of iron(III) chloride hexahydrate, 1.713 g of ferrous(II) sulfate heptahydrate, and 6 g of urea were dissolved in 50 mL of deionized water in a three-necked flask with a connected reflux condenser. Then, 15 ml of treated suspension rGO was added into the flask. After that, 50 mL of deionized water was added to the solution of rGO and iron salts with constant mixing on a magnetic stirrer at 300 rpm for 10 min. The solution was next heated at 115 °C for 18 h with stirring at 800 rpm and then cooled to room temperature. A precipitate was magnetically separated and washed with deionized water until neutral pH was reached. Then, the sample was dried at 35 °C in a convection oven for 2 days. The synthesis of magnetite can be described by the following reactions
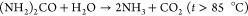
1
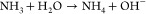
2
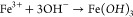
3
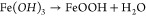
4
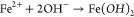
5
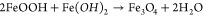
6

At the beginning of the procedure, yellow precipitate was observed, indicating the formation of Fe(OH)_3_ as a consequence of Fe^3+^ hydrolysis. After 8 h, the color of the reaction system began to darken, and after 10 h the color turned black, which indicated the emergence of Fe_3_O_4_. When a solution containing Fe^2+^, Fe^3+^, and dissolved urea is heated to >70 °C, urea decomposes into CO_2_ and NH_3_ (eq 1). Under reflux conditions, CO_2_ leaves the system and therefore only NH_3_ reacts with water to form hydroxyl ions (eq 2). With increasing pH, Fe(OH)_3_ precipitates first (eq 3). Fe(OH)_3_ next converts to FeOOH (eq 4), known as goethite. Once enough hydroxyl ions have formed, Fe(OH)_2_ begins to precipitate (eq 5). Next, magnetite is generated from the available FeOOH and Fe(OH)_2_ nuclei (eq 6).

### 2.3 Fabrication of PHB/Fe_3_O_4_–rGO Composites by Electrospinning

Dry PHB polymer powder (natural origin, Sigma-Aldrich) was dissolved in chloroform (CHCl_3_, Sigma-Aldrich) to achieve a concentration of 6 wt % and served as a control. For obtaining PHB/Fe_3_O_4_–rGO composites, 8 wt % of Fe_3_O_4_–rGO was dispersed in chloroform and sonicated using an ultrasound homogenizer Scientz-IID (Ningbo SCienta Biotechnology Co. Ltd., China) for 2 h at room temperature. Then, 6 wt % of PHB powder was introduced into the Fe_3_O_4_–rGO suspension and placed on the magnetic stirrer rotating at a speed of 400 rpm at 60 °C for 2 h for incubation. After that, pure PHB and PHB/Fe_3_O_4_–rGO composite scaffolds were prepared by the electrospinning technique using the following parameters:

- Pure PHB with a 27-gauge needle (*d* = 0.2 mm), collector rotation speed: 200 rpm, voltage: 5.2 kV, flow rate: 0.3 mL/h, and needle–collector distance: 10 cm (PHB_G27_).
- Pure PHB with a 21-gauge needle (*d* = 0.51 mm), collector rotation speed: 200 rpm, voltage: 9.1 kV, flow rate: 0.85 mL/h, and needle–collector distance: 14 cm (PHB_G21_).
- PHB/Fe_3_O_4_–rGO with the 27G needle (*d* = 0.2 mm), collector rotation speed: 200 rpm, voltage: 7.2 kV, flow rate: 0.32 mL/h, and needle–collector distance: 10 cm (PHB/Fe_3_O_4_–rGO_G27_).
- PHB/Fe_3_O_4_–rGO with the 21G needle (*d* = 0.51 mm), collector rotation speed: 200 rpm, voltage: 10.6 kV, flow rate: 0.85 mL/h, and distance from the collector to the needle: 14 cm (PHB/Fe_3_O_4_–rGO_G27_).

### 2.4 Characterization of Fe_3_O_4_–rGO Composite Fillers and Electrospun PHB/Fe_3_O_4_–rGO Scaffolds

The morphology of the Fe_3_O_4_–rGO composite fillers and electrospun fibrous scaffolds was examined under a scanning electron microscope (Quanta 600; Thermo Fisher, Japan). Scanning electron microscopy (SEM) was performed three times at different magnifications for each sample. Diameter distributions of the particles and fibers were calculated with the ImageJ software.

Phase composition was characterized by X-ray diffraction (XRD) analysis on a Shimadzu XRD 7000S diffractometer (Japan) equipped with a high-speed 1280-channel OneSight detector using Cu Kα radiation (λ = 1.5406 Å) at a scan rate of 4°/min and a step size of 0.02° in 2θ Bragg–Brentano geometry. The XRD patterns were recorded in the 2θ range from 5 to 80° thrice. Crystallite size (*D*_*hkl*_) was estimated according to Scherrer’s equation
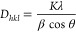
7where λ is the X-ray wavelength, β is the peak width at half height, *K* is a dimensionless particle shape factor (usually set to 0.9^46^), and θ is the diffraction angle.

Raman spectra and optical photographs were obtained using an NT-MDT microscope (Russia) equipped with a 100× objective. A semiconductor laser at a wavelength of 633 nm with a maximum power of 50 mW was utilized. To prevent heating of the sample and phase transformations, only 1% of the laser power was applied. All the samples of every studied group were examined in five different points.

Mechanical properties of the electrospun fibrous scaffolds were evaluated under ambient conditions on an Instron 3369 universal testing machine (United States) at a loading rate of 1 mm/min. Samples with an average thickness of 150 μm were cut out in a rectangular shape with a length of 50 mm and a width of 10 mm. Mean stress–strain curves for each sample were constructed via averaging of six samples. Young’s moduli were extracted as the slope of the linear section of the curves. Statistical analysis of the data was performed by one-way ANOVA in the Origin software.

Differential scanning calorimetry (DSC) was performed to evaluate crystallinity alterations in PHB scaffolds doped with rGO and Fe_3_O_4_ using a DSC Q2000 instrument (USA). DSC analysis of the fabricated scaffolds with a mass of ∼5 mg was carried out in an aluminum pan, and the temperature was varied from 75 to 225 °C at a heating rate of 10 °C/min in a nitrogen atmosphere. DSC analysis was performed three times for each sample. The degree of crystallinity (*X*_c_) of the fabricated scaffolds was calculated via the formula.^47,48^
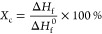
8where Δ*H*_f_ is the heat of fusion (J/g) and Δ*H*_f_^0^ is the heat of fusion of 100% crystalline PHB, equivalent to 146 J/g.^49^

Magnetic properties of Fe_3_O_4_–rGO composite fillers and electrospun fibrous scaffolds were investigated at a temperature of 300 K in an external pulsed magnetic field of 0–6.5 kOe on a pulsed magnetometer. All the samples of every studied group were investigated thrice. The measurements were carried out according to a technique described elsewhere.^50^

To characterize the surface chemistry of the scaffolds, X-ray photoelectron spectroscopy (XPS) was performed using a Thermo Fisher Scientific XPS NEXSA spectrometer (Thermo Fisher Scientific, Waltham, MA, United States) with a monochromated Al Kα Alpha X-ray source operating at 1486.6 eV. The XPS spectra were acquired from a 400 μm^2^ surface area of scaffolds three times (survey spectra: pass energy of 200 eV and energy resolution of 1 eV and high-resolution spectra: pass energy of 50 eV and energy resolution of 0.1 eV). A flood gun was used to compensate the charge.

The magnetic phase, surface electric potential, and piezoelectric response of individual polymer fibers at the nanoscale were investigated by magnetic force microscopy (MFM), Kelvin probe force microscopy (KPFM), and piezoresponse force microscopy (PFM), respectively, by means of a scanning probe microscope [Ntegra Aura Atomic Force Microscope (AFM); NT-MDT, Russia] equipped with an external HF2LI Lock-in Amplifier (Zurich Instruments, Switzerland). For KPFM measurements, conductive Cr/Pt-coated Multi75-G cantilevers (Budget sensors, Bulgaria) with a spring constant of 3 N/m and a resonance frequency of 75 kHz were used. Surface potential of the fibers was determined with a two-pass technique under a noncontact regime at the fundamental resonance of the cantilever. To minimize a parasitic electrostatic contribution in the PFM measurements,^51,52^ hard conductive Cr/Pt-coated Tap 190-G cantilevers (Budget Sensors, Bulgaria) with a high spring constant (48 N/m) and resonance frequency of 190 kHz were utilized, and external DC voltage was applied to compensate the surface potential. Piezoelectric response of the fibers was recorded in the contact mode at a frequency of 21 kHz and AC excitation voltages of 3, 6, and 9 V. All investigations were carried out thrice for every group of samples.

Structure and microstructure of the samples were assessed by high-resolution transmission electron microscopy (HR-TEM) using a ThemisZ electron microscope (Thermo Fisher Scientific, USA) operated at an accelerating voltage of 200 kV. A microscope is equipped with a corrector of spherical aberrations, thus providing a maximum lattice resolution of 0.06 nm, and with a SuperX energy-dispersive spectrometer (Thermo Fisher Scientific, USA). Images were captured by a Ceta 16 CCD sensor. For electron-microscopy analyses, samples were deposited on perforated carbon substrates attached to aluminum grids with the help of an ultrasonic dispersant. All the samples of every studied group were examined three times.

## 3 Results and Discussion

### 3.1 Characterization of the Fe_3_O_4_–rGO Composites

**Figure 1:**
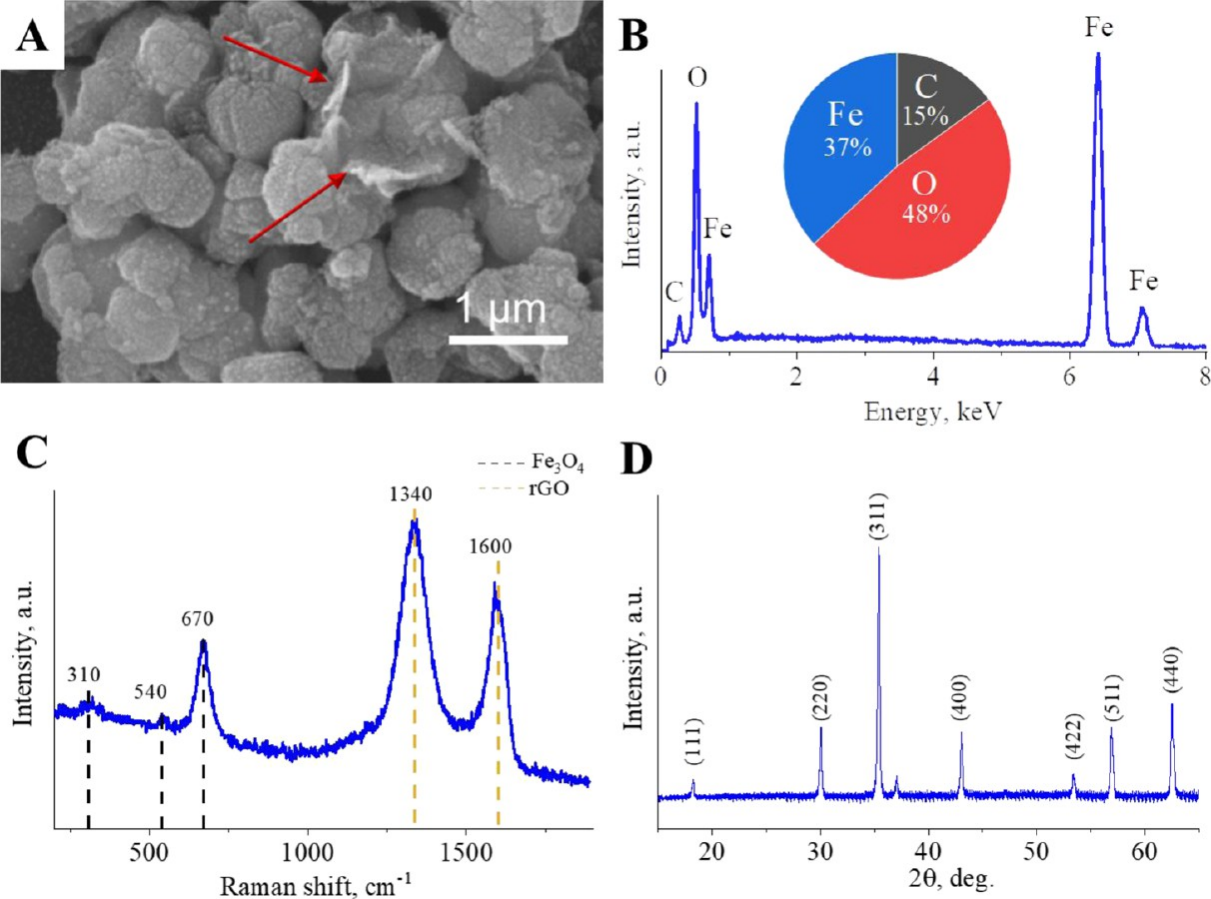
SEM image (A), EDX results (B), Raman spectrum (C), and XRD pattern (D) of the Fe_3_O_4_–rGO composites.

Figure 1A shows a SEM image of the Fe_3_O_4_–rGO composite formed by coprecipitation of iron salts on the surface of rGO. The Fe_3_O_4_–rGO composite consists of large agglomerates with a size of 0.97 ± 0.27 μm (mean ± SD). The internal content of the agglomerates represents sheets of rGO (marked with red arrows in Figure 1A). The semi-quantitative EDX analysis revealed the presence of 15 at. % of carbon, 48 at. % of oxygen, and 37 at. % of iron (Figure 1B) in the Fe_3_O_4_–rGO composite. In addition, the Fe/O ratio (0.77) in the Fe_3_O_4_–rGO composite obtained by EDX analysis is very close to that of stoichiometric magnetite (0.75).

It is likely that the synthesis of the Fe_3_O_4_–rGO composite was facilitated by the chemisorption mechanism. Positively charged iron ions (Fe^2+^ and Fe^3+^) bound to the negatively charged rGO surface, which acted as a nucleation center owing to the electrostatic interaction. The large surface area (100 m^2^/g), increased interlayer spacing, and evenly distributed active sites are helpful for efficient anchoring of positive iron ions and subsequent growth of Fe_3_O_4_ particles, as described elsewhere.^53,54^

Figure 1D shows that the Fe_3_O_4_–rGO composite yielded XRD peaks at 30.35, 35.63, 43.49, 53.56, 57.12, and 62.81° corresponding to (220), (311), (400), (422), (511), and (440) crystal planes of magnetite (PDF card 01-080-6403). A typical rGO reflection at 24.56° was absent in the synthesized Fe_3_O_4_–rGO composite.^55^ The mass ratio of the initial components used in the synthesis plays a key role in the preparation of a Fe_3_O_4_–rGO composite. It is reported that the peaks of rGO in XRD patterns appear only at a relatively high rGO content in Fe_3_O_4_–rGO composites.^56^

The identification of magnetite and maghemite (γ-Fe_2_O_3_) by XRD analysis alone is quite a challenging task because these materials have spinel-type structures and very similar lattice unit parameters (0.8350 nm for γ-Fe_2_O_3_ and 0.8396 nm for Fe_3_O_4_).^57^ Therefore, Raman spectroscopy was chosen to confirm the phase composition of the obtained iron oxide particles. According to the results, magnetite/rGO composites possess three characteristic vibrational modes of the magnetite phase: E_g_, T_2g_(3), and A_1g_ at 310, 540, and 670 cm^–1^, respectively.^58^ The presence of the band at 670 cm^–1^ (A_1g_) is due to the symmetrical displacement of oxygen atoms in the FeO_4_ tetrahedral group along the [111] direction. Vibrational mode E_g_ at 310 cm^–1^ characterizes the symmetrical bending of oxygen with respect to iron in the tetrahedral void.^59^ The T_2g_(3) mode arises due to the asymmetric stretching of iron and oxygen that takes place due to the displacement of oxygen and iron cations at tetrahedral sites. Two peaks characteristic of rGO—at 1340 and 1600 cm^–1^—belonging to D and G bands, respectively, were observed in the Raman spectrum of the newly developed Fe_3_O_4_–rGO composite.^60^ The G band corresponds to the first-order scattering of the E_2g_ mode for sp^2^-carbon domains, and the pronounced D band is related to structural defects of a carbon lattice.^61^ In contrast to the Raman spectrum of rGO (Figure S1), for the Fe_3_O_4_–rGO composite, the D band is more intense than the G band and this effect is related to the formation of sp^3^-hybridized bonds.^62^ This finding is confirmed by a comparison of intensity ratios of D and G bands (*I*_D_/*I*_G_), which can be used to describe defect density in graphene-based materials.^63^ The *I*_D_/*I*_G_ ratios of rGO and Fe_3_O_4_–rGO composites were calculated: 1.08 and 1.36, respectively. The higher *I*_D_/*I*_G_ ratio for the composite confirms that more defects and imperfections are present in Fe_3_O_4_–rGO than in the initial rGO. This difference may be explained by greater distances between the layers and a higher degree of exfoliation due to the presence of Fe_3_O_4_ particles.^62^ Besides, no peaks of other iron oxides were found, thus confirming pure-phase magnetite in the Fe_3_O_4_–rGO composites.

**Figure 2:**
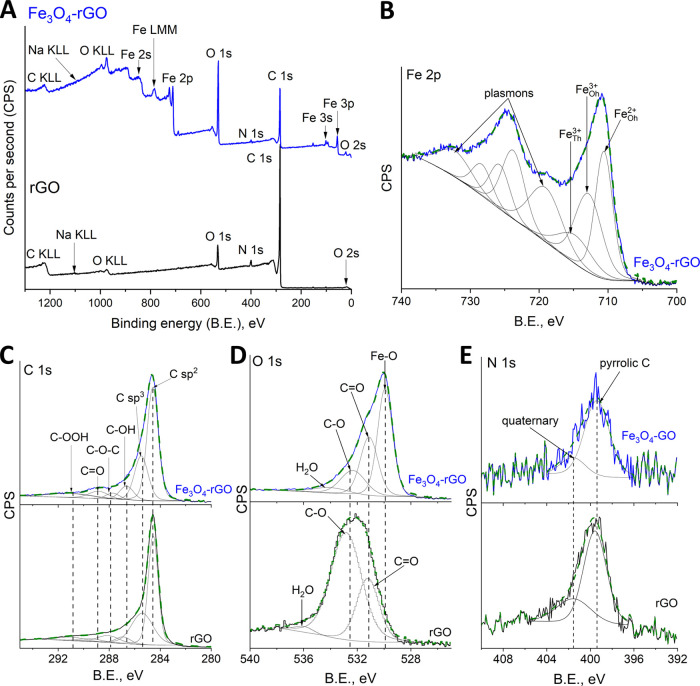
Survey (A) and high-resolution (B–E) XPS spectra of Fe 2p (B), C 1s (C), O 1s (D), and N 1s (E) regions for the synthesized Fe_3_O_4_–rGO composite and commercial rGO.

Surface composition of the synthesized Fe_3_O_4_–rGO composite was evaluated by XPS. All expected elements, such as C, O, N, and Fe, were successfully detected in pristine rGO and newly developed Fe_3_O_4_–rGO particles (Figure 2A). Relative atomic concentrations of C (78 at. %), O (18 at. %), and N (4 at. %) are well consistent with the manufacturer’s information. The formation of Fe_3_O_4_ lowered C and N concentrations down to 20 and 1 at. %, respectively. Furthermore, a high Fe content (50 at. %) was registered, as was a higher content of O, up to 29 at. %. Meanwhile, a fitting of the Fe 2p region for the Fe_3_O_4_–rGO composite revealed the presence of Fe^2+^ and Fe^3+^ ions corresponding to octahedral (denoted as Oh) and tetrahedral (denoted as Th) interstitial sites from the cubic spinel-type structure (Figure 2B).^64^ Meanwhile, the deconvolution of the C 1s region for the newly developed Fe_3_O_4_–rGO composite uncovered all typical peaks of rGO (Figure 2C) such as C sp^2^, C sp^3^, C–OH, C–O–C, C=O, and C–OOH.^64,65^ Aside from these results, the analysis of the O 1s region indicated the presence of a Fe–O peak from magnetite as well as functional groups C–O and C=O (Figure 2D).^65^ The fitting of the N 1s region revealed two typical peaks from rGO corresponding to pyrrolic C and quaternary C for pristine rGO and Fe_3_O_4_–rGO composites (Figure 2E).^65^ To sum up, the XPS analysis confirmed the formation of magnetite particles on rGO flakes.

It is worth mentioning that for the synthesized Fe_3_O_4_–rGO composite, the main contribution to the C 1s region is expected from rGO. As compared to pristine rGO, the in situ formation of Fe_3_O_4_ on the surface of rGO sheets gave rise to the C 1s region, for example, by raising the number of functional groups C=O (Figure 2C). At the same time, the O 1s region also manifested an increase in the content of polar functional group C=O as compared to the C–O group, which is dominant in pristine rGO according to our results (Figure 2D) and the literature.^66^

**Figure 3:**
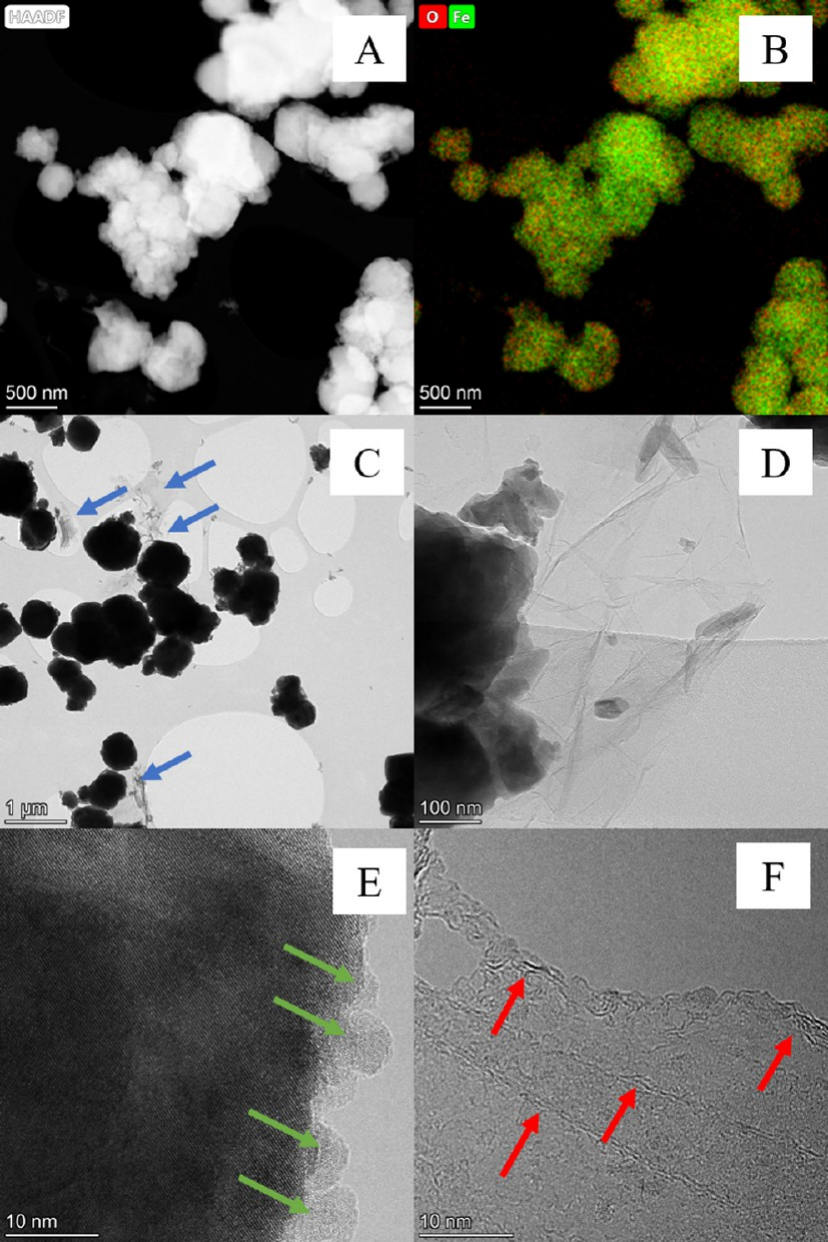
HAADF-STEM image (A), mixed-color elemental mapping (B), and TEM image (C) of the Fe_3_O_4_–rGO composite; TEM image illustrating rGO morphology (D), HR-TEM image depicting the Fe_3_O_4_ crystal structure (E), and HR-TEM image illustrating the rGO crystal structure (F).

To reveal the morphology and structure of the Fe_3_O_4_–rGO composites, a side section was examined by TEM analysis. The STEM image and corresponding EDX mapping showed uniform elemental distribution and ∼500 nm particles consisting of smaller particles with a size of ∼200 nm (Figure 3A,B). In the TEM images in Figure 3C, the gray transparent parts (indicated by blue arrows) represent rGO sheets, while the black-rounded parts denote Fe_3_O_4_ particle agglomerates. Fe_3_O_4_–rGO composites of the semispherical shape ranging in size from 0.7 to 1.1 μm are visible. Figure 3D illustrates the morphological characteristics of the rGO sheets. The image shows graphite-like structures and iron oxide particles on the surface; it should be pointed out that the thickness of these layers varies, as follows from the thickness of the side sections. Some regions of the rGO sheets are covered with Fe_3_O_4_ particles, while other regions seen via TEM are not. Therefore, we can assume that on the surface of the rGO flakes, the Fe_3_O_4_ particles are not distributed uniformly. Fe_3_O_4_ microparticles have nanofeatures on the surface as a consequence of the growth mechanism. These results are in good agreement with the SEM findings. A typical HR-TEM image of a single Fe_3_O_4_ particle is given in Figure 3E: the examined particle has an interplanar distance of 2.9 Å, which is very close to the *d*_220_ plane of magnetite.^67^ On the surface of this particle, smaller particles are visible (pointed out by green arrows) also belonging to the magnetite phase according to the observed interplanar distances. As mentioned above, rGO possesses a sheet-like structure with a smooth surface and wrinkled edges (Figure 3E). The image clearly shows graphite layers and an edge with surface defects (indicated by red arrows).

It must be mentioned that in an aqueous medium, Fe_3_O_4_ particles undergo agglomeration with neighboring Fe_3_O_4_ particles owing to their magnetic nature. On the other hand, rGO layers are also subject to irreversible aggregation and repacking due to π–π interactions between neighboring sheets, and all this together reduces the active surface area and adsorption capacity of rGO while reducing the reactivity of Fe_3_O_4_ particles and rGO sheets. To overcome the above limitations, Fe_3_O_4_ particles were nucleated and grown directly on rGO active centers. rGO can provide a large surface area for the growth of Fe_3_O_4_ particles with a uniform distribution and prevent irreversible agglomeration of the particles even in an aqueous environment.

When a solution containing Fe^2+^ and Fe^3+^ and dissolved urea is heated to >85 °C, urea decomposes into CO_2_ and NH_3_ (eq 1). OH^–^ ions are gradually released during the hydrolysis of urea and interact with Fe^3+^ and Fe^2+^, thereby producing Fe(OH)_3_ and Fe(OH)_2_, respectively. Under reflux conditions, CO_2_ leaves the system and, therefore, only NH_3_ reacts with water to form OH^–^ (eq 2). As pH increases, iron hydroxide Fe(OH)_3_ precipitates first (eq 3). Fe(OH)_3_ then changes to FeOOH (eq 4), known as goethite, which is shaped like a needle.

**Figure 4:**
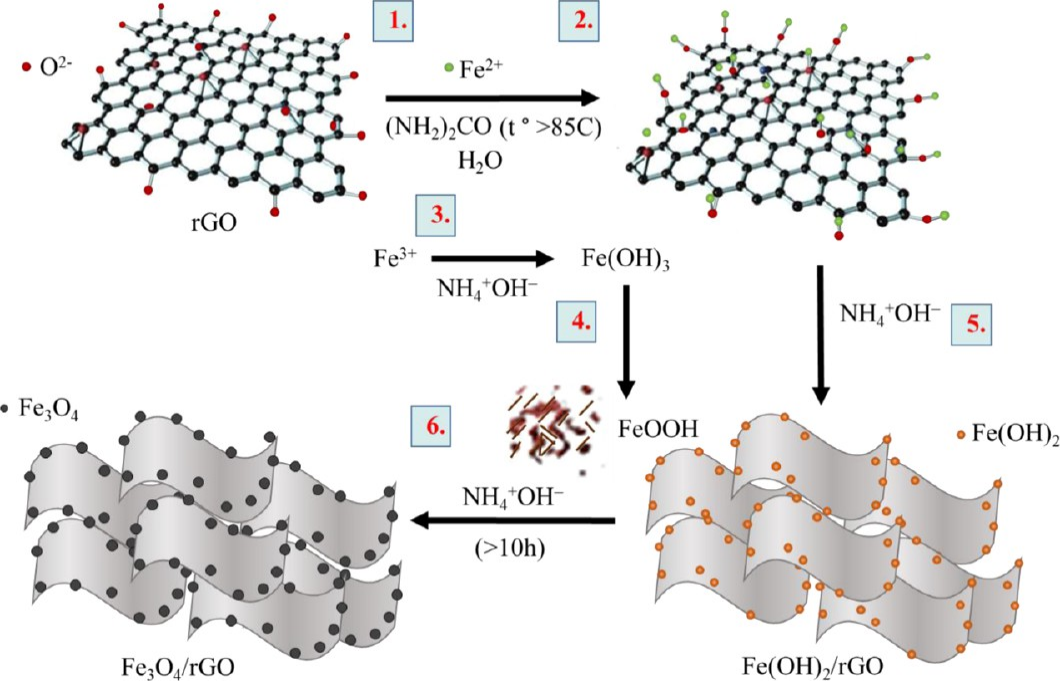
Proposed reaction mechanism underlying the synthesis of the Fe_3_O_4_–rGO composite.

By analogy with the mechanism proposed in refs (53) and (68), it can be theorized that due to a strong coordination interaction between Fe^2+^ ions and residual oxygen functional groups of rGO, Fe^2+^ ions are fixed on its surface during the mixing (eq 4). Next, after the formation of a sufficient amount of hydroxyl ions in the reaction medium, Fe(OH)_2_ comes into being on the surface of rGO (eq 5). Because FeOOH and Fe(OH)_2_ nuclei are present, the particles gradually grow and transform into homogeneous Fe_3_O_4_ (eq 6) (Figure 4).

At the beginning of the reaction, yellow precipitates are visible, indicating the formation of Fe(OH)_3_ as a product of hydrolysis of the Fe^3+^ salt. After 8 h, the color of the reaction system begins to darken; after 10 h and later, the color becomes black, pointing to the formation of Fe_3_O_4_. As a consequence of the presence of a large number of nucleation centers, the rGO surface turned out to be covered with a dense layer consisting of the Fe_3_O_4_ particles, which is gradually formed during 18 h of the synthesis under the conditions of urea decomposition.

### 3.2 Characterization of the PHB/Fe_3_O_4_–rGO Scaffolds

**Figure 5:**
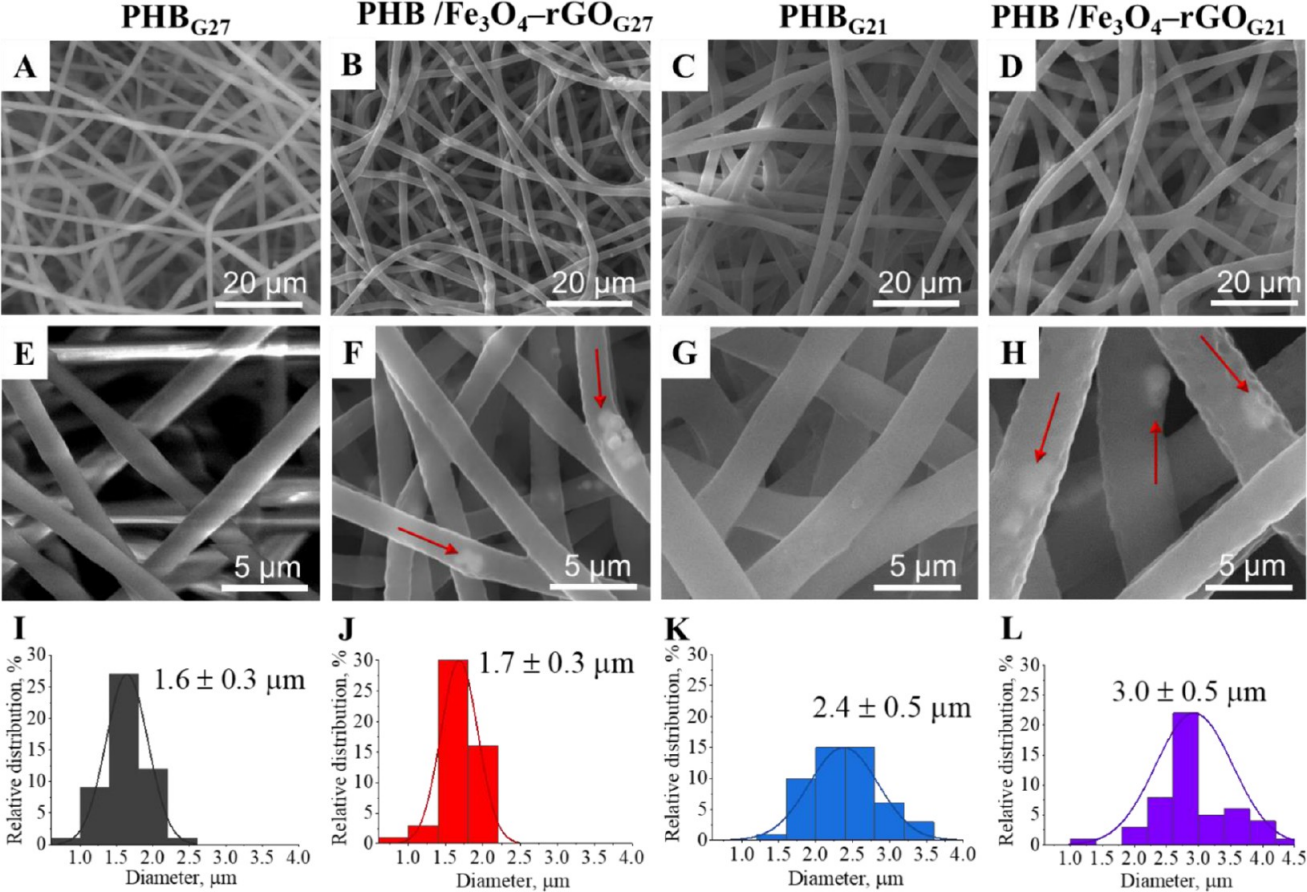
SEM images (A–H) and fiber diameter distributions (I–L) of the PHB/Fe_3_O_4_–rGO composite scaffolds.

SEM was performed to study the morphology of the scaffolds doped with the composite Fe_3_O_4_–rGO fillers. Figure 5 shows SEM images and relative fiber diameter distributions for all the fabricated scaffolds. As compared to pure PHB scaffolds, the fibers of the obtained composite scaffolds have small defects, contain a small number of agglomerates of the Fe_3_O_4_–rGO composite protruding to the periphery, and morphologically differ from defect-free and smooth fibers of pure PHB scaffolds. As depicted in the figure, Fe_3_O_4_–rGO agglomerates are less pronounced for PHB/Fe_3_O_4_–rGO_G21_ scaffolds owing to the larger fiber diameter. Overall, the fibers do not have defects, thereby indicating the correct selection of parameters for preparing the solutions as well as good stability of the electrospinning process.

The diameters of pure PHB scaffolds proved to be 1.6 ± 0.3 and 2.4 ± 0.5 μm for PHB_G27_ and PHB_G21_, respectively. The fiber diameters of the composite scaffolds are 1.7 ± 0.3 and 3.0 ± 0.5 μm for PHB/Fe_3_O_4_–rGO_G27_ and PHB/Fe_3_O_4_–rGO_G21_ scaffolds, respectively. It should be noted that the greater diameter of the needle used in the electrospinning process enlarged the average diameter of the fibers; this effect can be attributed to a change in the electrospinning parameters (viscosity, surface tension, and electrical conductivity of the solution), whereas the addition of 8 wt % of the Fe_3_O_4_–rGO composite fillers only insignificantly affected the average fiber diameter.

**Figure 6:**
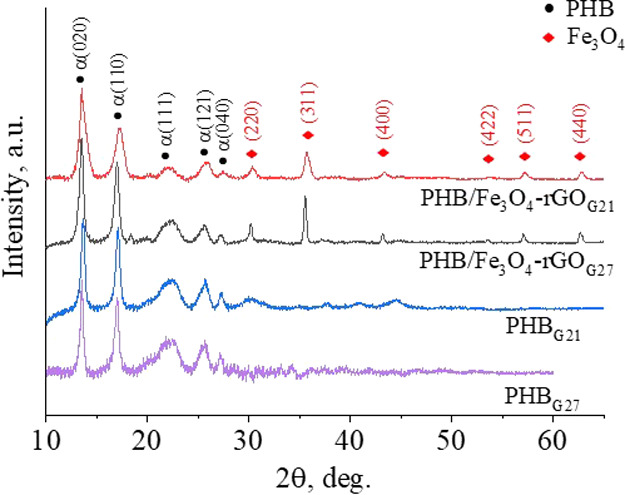
XRD patterns of pure PHB and PHB/Fe_3_O_4_–rGO composite scaffolds. Crystal planes of PHB and Fe_3_O_4_ are marked as (•) and (⧫), respectively.

The XRD patterns (Figure 6) of all the scaffolds contain typical characteristic peaks of the α-phase of PHB at 13.6, 16.9, 22.4, 25.5, 26.9, and 19.9° corresponding to (020), (110), (111), (121), (040), and (021) crystal planes of PHB (ICDD PDF card no. 00-068-1411). Reflections at 30.35, 35.63, 43.49, 53.56, 57.12, and 62.81° corresponding to (220), (311), (400), (422), (511), and (440) crystal planes of magnetite were registered for PHB/Fe_3_O_4_–rGO scaffolds. At the same time, no rGO reflections were detectable in PHB/Fe_3_O_4_–rGO composite scaffolds. Nonetheless, it was revealed that the incorporation of the Fe_3_O_4_–rGO composite diminished crystallite sizes of PHB in (020) and (110) directions (Table 1). Of note, different needle diameters used in the electrospinning had no significant impact on the crystallite size of pure PHB scaffolds. On the contrary, in the case of composite PHB/Fe_3_O_4_–rGO scaffolds, the needles with a smaller diameter during the electrospinning decreased the crystallite size of the polymer, and this phenomenon is related to the volume of the polymer passing through the needle. Fe_3_O_4_–rGO composite fillers limited the lamellae growth and lowered the crystallization degree; this effect is also attributable to reduced volume of the polymer solution passing through the needle.

**Table 1:** Crystallite Size of Pure PHB and PHB/Fe_3_O_4_–rGO Composite Scaffolds

	crystallite size, nm	
sample	(020)	(110)
PHB_G21_	20	15
PHB/Fe_3_O_4_–rGO_G21_	14	16
PHB_G27_	19	17
PHB/Fe_3_O_4_–rGO_G27_	8	10

**Figure 7:**
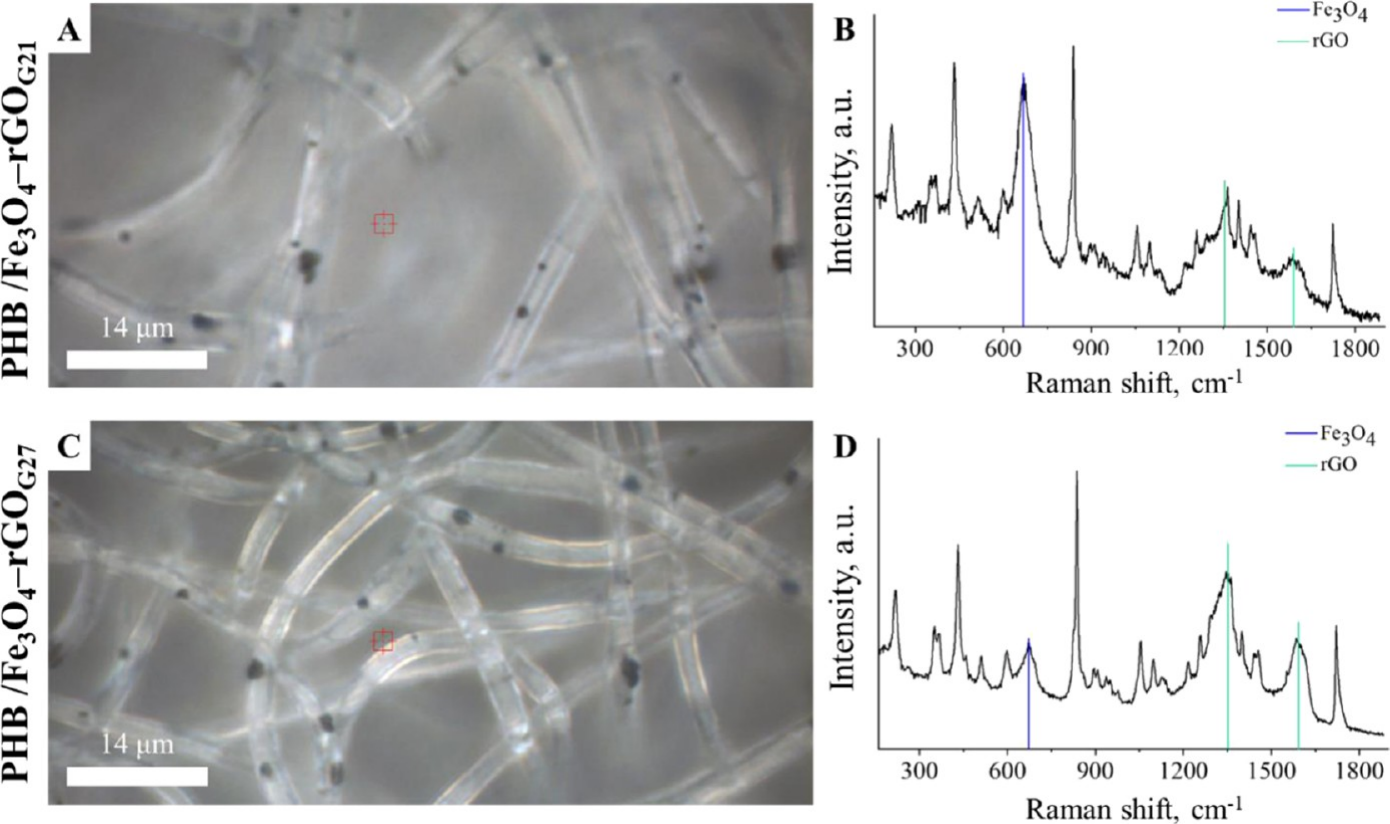
Optical micrographs (A,C) and Raman spectra (B,D) of PHB/Fe_3_O_4_–rGO composite scaffolds.

Figure 7 presents optical photographs and corresponding Raman spectra of the PHB/Fe_3_O_4_–rGO composite scaffolds. Raman shifts’ and bands’ assignments for PHB are summarized in Table 2. For both PHB/Fe_3_O_4_–rGO_G21_ and PHB/Fe_3_O_4_–rGO_G27_ scaffolds, an additional peak at 670 cm^–1^ (Figure 7B,D) was registered corresponding to Fe–O symmetric stretching vibrations of magnetite. Despite the peaks of PHB and magnetite, two additional characteristic peaks of rGO—at 1340 and 1600 cm^–1^—assigned to D and G bands, respectively, were observed too.

**Table 2:** Raman Shifts and Corresponding Assignments of the Bands for PHB^69^

Raman shift, cm–^1^	assignment
1725	C=O stretching vibrations (crystalline phase)
1460	CH_3_ asymmetric bending vibrations
1443	CH_2_ bending vibrations
1402	CH_3_ symmetric bending vibrations
1365	CH bending vibrations and CH_3_ symmetric bending vibrations
1295	CH bending vibrations
1261	C–O–C stretching vibrations and CH bending vibrations
1220	C–O–C asymmetric stretching vibrations
1101	C–O–C symmetric stretching vibrations
1058	C–O stretching vibrations
953	C–C stretching vibrations and CH_3_ rocking bending vibrations
841	C–COOstretching vibrations
691	C=O bending vibrations (in-plane)
680	C=O bending vibrations (out-of-plane)
598	C–CH_3_ and CCO bending vibrations
510	C–CH_3_ and CCO bending vibrations
367	C–CH_3_ and CCO bending vibrations
351	C–CH_3_ and CCO bending vibrations
222	CH_3_ torsion bending vibrations

**Figure 8:**
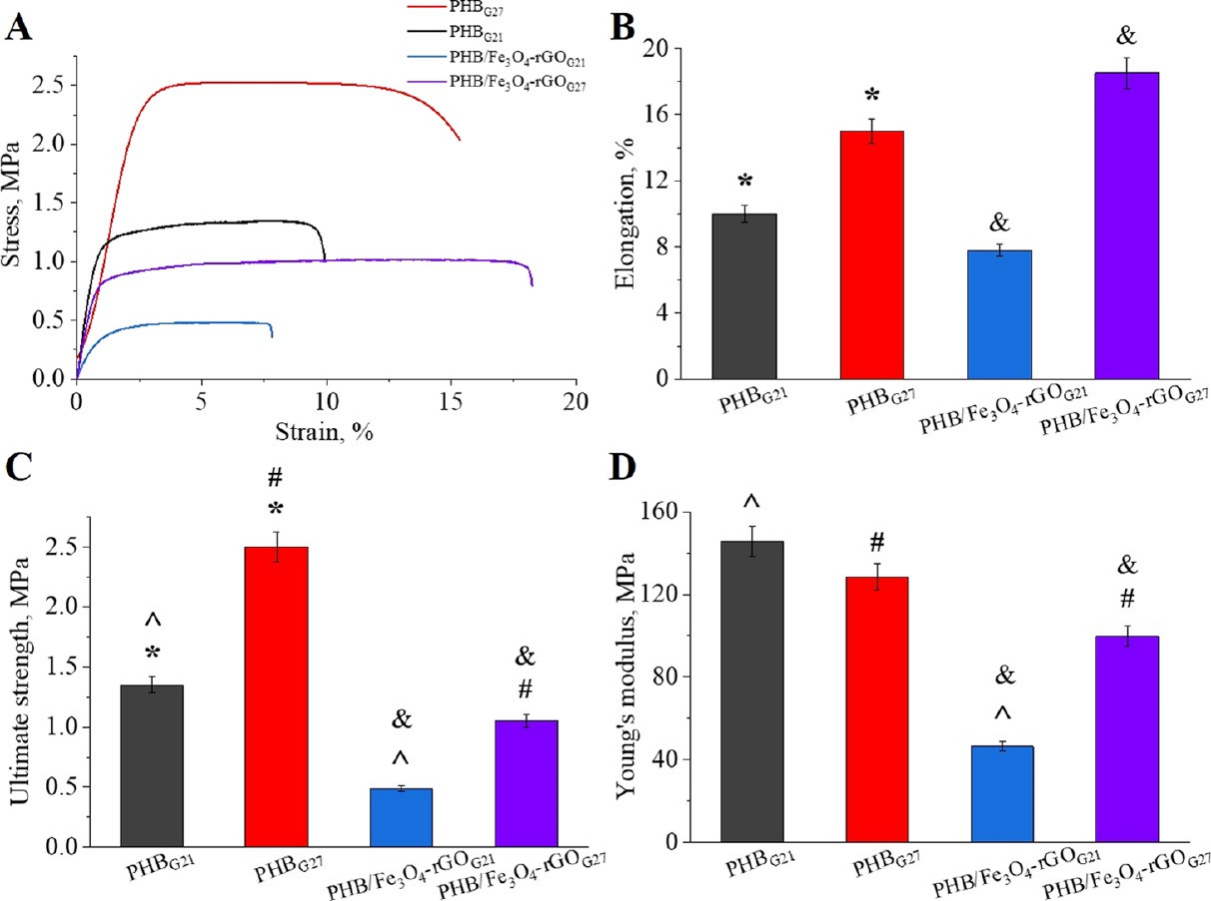
Stress–strain curves (A), elongation at break (B), ultimate tensile strength (C), and Young’s modulus (D) of the PHB/Fe_3_O_4_–rGO composite scaffolds. The symbols above the bars denote significant differences (*p* < 0.05) between PHB_G21_ and PHB_G27_ (*), between PHB_G21_ and PHB/Fe_3_O_4_–rGO_G21_ (^), between PHB_G27_ and PHB/Fe_3_O_4_–rGO_G27_ (#), and between Fe_3_O_4_–rGO_G21_ and Fe_3_O_4_–rGO_G27_ (&).

Scaffolds for tissue engineering should have sufficient mechanical strength to provide temporary support matching mechanical properties of the host tissue as closely as possible to withstand in vivo loading and stress conditions.^70^ To evaluate the influence of the fiber diameter and of incorporation of the Fe_3_O_4_–rGO filler on mechanical performance of the PHB scaffolds, tensile tests were performed. Typical stress–strain curves and computed physico-mechanical properties of pure and composite PHB scaffolds are displayed in Figure 8. As presented in Figure 8B, elongation at break went up after the decrease in the fiber diameter in both pure and composite scaffolds. In pure PHB scaffolds, elongation at break increased from 10 ± 1.5 to 15 ± 3.0%, whereas in the composites, this parameter improved even more markedly: from 7.8 ± 2.6 to 18.5 ± 5.7%. The enhanced ductility of finer fibers is well consistent with the literature^23,24^ and can be ascribed to a better ability of such fibers to absorb a considerable amount of energy before failure. Ramier et al.^23^ have prepared electrospun PHB nanofibers incorporating hydroxyapatite nanoparticles and documented an increase in elongation at break from 7.27 ± 0.49 to 12.48 ± 1.57% upon a reduction in the fiber diameter from 950 ± 160 to 640 ± 80 nm. Ultimate strength (Figure 8C) showed behavior similar to that of elongation at break. Thinner fibers fabricated using the 27G needle possess ultimate strengths of 2.50 ± 0.27 and 1.05 ± 0.18 MPa for pure and composite scaffolds, respectively, which are more than twice as high as those of PHB_G21_ and PHB/Fe_3_O_4_–rGO_G21_ fibers. We can conclude that the decrease in the fiber diameter from 2.4 ± 0.5 to 1.6 ± 0.3 μm in pure PHB and from 3.0 ± 0.5 to 1.7 ± 0.3 μm in composite PHB/Fe_3_O_4_–rGO scaffolds improves the scaffolds’ mechanical strength including elongation at break and ultimate tensile strength.

The addition of Fe_3_O_4_–rGO fillers to PHB scaffolds lowered ultimate strength from 1.35 ± 0.10 to 0.49 ± 0.15 MPa and from 2.50 ± 0.27 to 1.05 ± 0.18 MPa for the 21G and 27G groups of fibers, respectively. Moreover, Young’s moduli are lower in the composite scaffolds than in pure ones in both 21G and 27G groups (Figure 8D), indicating a stiffness decrease. Ghorbani et al. have noticed that the introduction of magnetite nanoparticles into polymeric structures gives higher toughness and improves strength.^20^ Besides, numerous research articles point to an enhancement of mechanical properties, including elastic modulus and tensile strength, after the addition of various carbon fillers.^17−19,25^ Ma et al. have detected significantly better tensile strength of PHBV upon supplementation with a small amount of multiwalled CNTs;^19^ however, a higher multiwalled-CNT content resulted in the emergence of agglomerates, which worsened mechanical properties of a PHBV–multiwalled nanotube nanocomposite. Similarly, incorporation of 0.5 wt % of CNTs improves the mechanical properties of an electrospun PHB scaffold because of a high degree of orientation of the filler in the nanofibers;^71^ nevertheless, as the concentration of the filler increased, the nanotubes aggregated, leading to a reduction in tensile strength and in elastic modulus of the scaffolds. What is more, the agglomeration enhanced van der Waals interactions between the CNTs and the walls of nanofibers.

The intrinsic mechanical performance of the composites compared to the pure PHB scaffolds may be ascribed to the aggregation of the magnetite particles and rGO flakes inside the fibers, as seen in SEM images (Figure 5). These agglomerates may act as stress concentration points and give rise to microcracks, worsening the composites’ mechanical strength.^72^ We suppose that lower concentrations of Fe_3_O_4_–rGO fillers could help to overcome aggregation and to achieve a more homogeneous distribution within the fibers, thus providing efficient load transfer and enhanced mechanical characteristics of the composite scaffolds. Moreover, to prevent aggregation, the surfaces of both magnetic particles and rGO could be functionalized in a different way, for example, by means of inorganic materials, small organic molecules, or macromolecules.^34,73^

**Figure 9:**
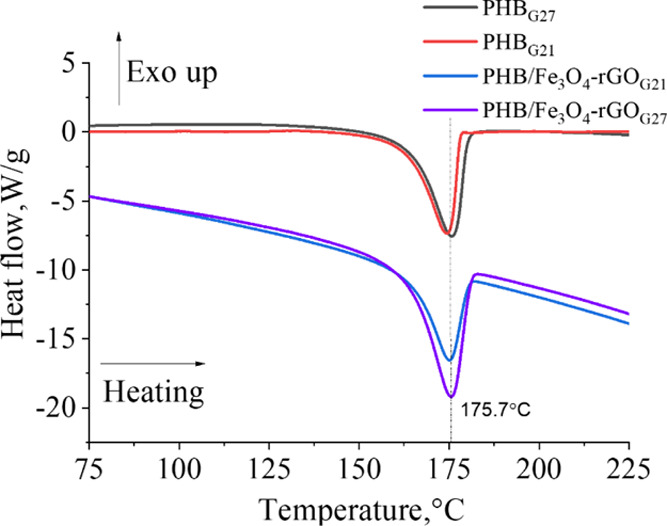
DSC curves of pure and composite PHB scaffolds.

The degree of crystallinity is known to have a tremendous impact on the degradation rates and mechanical properties of PHB.^26−28^ Therefore, it is crucial to understand the effect of the fiber diameter and of the addition of the filler on the degree of crystallinity of PHB scaffolds. In this regard, the DSC analysis of pure and composite scaffolds with various fiber diameters was conducted next (Figure 9). The calculated degree of crystallinity and the detected melting temperature of all the scaffolds are summarized in Table 3. A single melting peak was noted in the region 174–176 °C for all the samples. Regarding the influence of the filler incorporation on the polymer’s melting temperature, in the 21G group, *T*_m_ increased from 174 to 176 °C. A similar result was reported in a study on Zr(OH)_4_/PHB composites,^74^ where the melting temperature of PHB went up from 166 to 167 and 169 °C for films with 0.05 and 0.1% of incorporated Zr(OH)_4_, respectively. This finding was explained as follows: the PHB crystals had different morphological characteristics but very similar sizes. Notably, the melting temperature of scaffolds with thinner fibers (obtained with the 27G needle) slightly declined from 176 to 175 °C upon the addition of the Fe_3_O_4_–rGO filler. This effect can be attributed to the considerable reduction in crystallite sizes (Table 1) owing to the hindered crystallite growth of the composite.^75^ A similar decrease in *T*_m_ (from 176 to 175 °C) was documented for the composite scaffolds when we employed the thinner needle, again, consistently with the diminished crystallite sizes (Table 1).

**Table 3:** DSC Results on Pure and Composite Scaffolds

material	*T*_m_, °C	*X*_c_, %
PHB_G21_	174	58
PHB_G27_	176	53
PHB/Fe_3_O_4_–rGO_G21_	176	51
PHB/Fe_3_O_4_–rGO_G27_	175	50

The degree of crystallinity showed considerable changes. First, let us consider the influence of the fiber diameter; in the pure PHB scaffolds, the degree of crystallinity diminished from 58 to 53% upon the reduction in the fiber diameter. A similar crystallinity decrease (though to a smaller extent) was observed in the composites fabricated with the thinner needle (PHB/Fe_3_O_4_–rGO_G27_ compared to PHB/Fe_3_O_4_–rGO_G21_). Such fiber size-dependent crystallinity was also revealed in a study on electrospun PLLA nanofibers,^42^ where the nanofibers with a fiber diameter of 30 nm manifested approximately 40% crystallinity, whereas 500 nm nanofibers had approximately 48% crystallinity.

Electrospinning parameters are known to influence the degree of crystallinity of polymers.^76,77^ High elongation and shear stresses affecting polymer chains within an electric field during electrospinning cause the macromolecular chains to align along the fiber axis, thereby leading to a high degree of molecular orientation in the fibers, which is directly proportional to the degree of crystallinity.^76^ Zhao et al. have suggested that the molecular orientation is induced by the electrostatic field and that the degree of crystallinity of electrospun fibers is greatly influenced by crystallization time.^77^ According to Avrami’s equation, the degree of crystallinity is an increasing function of crystallization time^29^
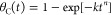
9where θ_C_(*t*) is the proportion of crystallized material at time *t*, *n* is the Avrami exponent, and *k* is the overall crystallization rate constant. Wu et al.^78^ have demonstrated high transient inhomogeneity of solvent concentration across a jet cross-section by modeling solvent evaporation from a polymer jet in electrospinning. They found that the simulated jet drying time shortens rapidly with a reduction in the initial jet diameter. Consequently, because thinner fibers have less time to crystallize, they have lower degrees of crystallinity.

In our recent study,^79^ we noticed a similar dependence of the degree of crystallinity on geometrical size, though in that case, *X*_c_ increased proportionally with greater thickness of solvent-cast PHB films. *X*_c_ was found to be 55, 57, and 61%, respectively, for 30, 60, and 100 μm thick films. In the present work, we can conclude that the crystallization of PHB may be limited by fiber thickness (i.e., diameter); this relation can be explained by the limited mobility of polymer chains owing to dimensional confinement (e.g., film size, thickness).^40,41,80^ It is noteworthy that several authors have stated that upon dimensional constraints (i.e., thinner fibers), crystal growth tends to be more anisotropic, where the crystals are forced to grow preferentially in the direction along the axis of the polymer fibers.^40,80^

It is worth mentioning the influence of the needle tip-to-collector distance (NTCD) on the scaffolds’ crystallinity because we chose different NTCD for each needle diameter. Increasing the NTCD extends the flight time of a solution jet.^81,82^ Given that molecular orientation can be facilitated by an electric field during electrospinning, if the flight time of the jet is extended, then it would be reasonable to say that the degree of crystallinity of PHB is higher at longer NTCDs. Accordingly, we observed higher *X*_c_ for the scaffolds fabricated by means of the thicker needle (PHB_G21_ and PHB/Fe_3_O_4_–rGO_G21_), for which optimal NTCD was found: 14 cm (whereas for the 27G group, it was 10 cm). From these findings, it can be deduced that the lower degree of crystallinity of the scaffolds fabricated with the thinner 27G needle may be attributed to four factors: (i) reduced time of crystallization owing to the rapid solvent evaporation and PHB solidification; (ii) shorter distance between the tip and collector; this parameter directly affects the duration of crystallization; (iii) constrained mobility of polymeric chains and spatially limited crystallization; and (iv) lower electrospinning voltage (see the Materials and Methods section) affording a lesser extent of molecular orientation. These results are consistent with the data from the mechanical tests because the more amorphous fibers with a smaller diameter manifested higher plasticity (i.e., elongation at break), as displayed in Figure 8B.

Notably, as compared to pure scaffolds, the degree of crystallinity of the composite ones decreased from 58 to 51% and from 53 to 50% for the 21G group and 27G group, respectively. Incorporation of various fillers into the polymer matrix may cause both an enhancement and reduction of a polymer’s crystallinity.^34^ On the one hand, fillers may act as nucleating agents when their concentration is relatively low and they are homogeneously distributed within the fibers.^31,34−37^ For example, the authors of ref (31) claim that hydroxyapatite nanoparticles promote the crystallization of PHB by acting as a nucleating agent during crystallization. By contrast, when the concentration of the nanoparticles is too high, they tend to form agglomerates, which restrain the mobility of polymer chains and hinder the crystallization.^32−36,38,39^ Ho et al. have demonstrated that the incorporation of 1 or 5 wt % of magnetite particles lowers the crystallinity of PHB and PHBV.^32^ This result was attributed to be a hindrance to the proper arrangement of polymer chains in the presence of magnetite. Furthermore, in ref (39), HA particles were found to act as additional nucleation sites, and small amounts of HA dramatically raised the crystallization rate relative to pure PHB. On the contrary, high HA contents (>20 wt %) clearly retarded the growth process. Wei et al.^36^ have prepared GO/poly(l-lactide) nanocomposites and found that the addition of modified GO promoted PLLA crystallization; however, when the filler content was too high, the aggregation of the filler hindered the proper arrangement of the PLLA chains, thereby having an adverse effect on the crystallization process, resulting in a slight decrease in crystallinity.

Accordingly, we believe that one of the key reasons for the diminution of the composites’ crystallinity is the aggregation of Fe_3_O_4_ and rGO particles observed in the SEM images (Figure 5). These agglomerates reduced the mobility of PHB chains and hindered their ordered arrangement. The constrained crystallization corresponds to diminished crystallite sizes in the (020) and (110) planes of the composite scaffolds as compared to the pristine ones (Table 1). In addition, the reduction in crystallinity correlates with lower ultimate tensile strength and Young’s modulus of the composites (Figure 8C,D).

**Figure 10:**
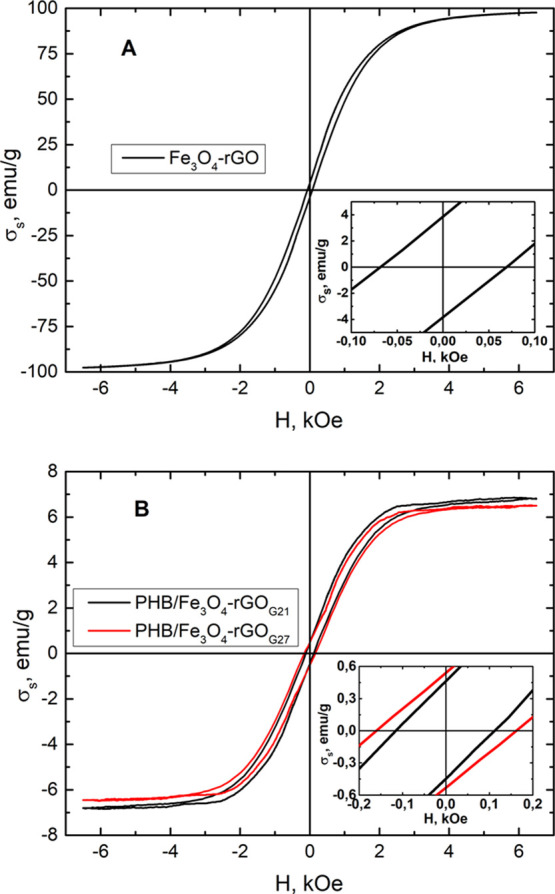
Magnetic hysteresis loops of the Fe_3_O_4_–rGO (A) composite and PHB/Fe_3_O_4_–rGO (B) scaffolds made with 27G and 21G needles. The insets are an enlarged view of the magnetization curves showing a coercive force for the samples.

Magnetic particles may act as magnetomechanical remote actuators to improve cell growth during the application of external magnetic fields.^83^ When investigators develop magnetic composites and polymer magnetic scaffolds for various biomedical applications, it is important to know the magnetic properties the scaffolds acquire as a consequence of the introduction of magnetite particles. The main magnetic characteristics include saturation magnetization (σ_s_), remanent magnetization (σ_r_), and coercive force (*H*_c_). The magnetic properties of the composites and polymeric scaffolds assayed here are listed in Table 4. Figure 10 presents magnetic hysteresis loops of the Fe_3_O_4_–rGO composite as well as those of PHB/Fe_3_O_4_–rGO scaffolds fabricated with 27G and 21G needles. Saturation magnetization of the magnetite particles used in the present study was evaluated in our previous work.^84^ The saturation magnetization of the Fe_3_O_4_–rGO composites is 96.27 ± 1.42 emu/g, which is lower than that of pure Fe_3_O_4_ owing to the presence of nonmagnetic rGO. Nonetheless, σ_s_ is rather high. The contribution to σ_s_ is mediated by a paraprocess and by defects on the surface layer of magnetite crystallites. Such defects can lead to the breakage of exchange bonds between ionic bonds at the tetrahedral position of the crystal lattice of magnetite, whose magnetic ions make a negative contribution to σ_s_.^85^ The coercive force (*H*_c_) for Fe_3_O_4_–rGO was found to be 60 ± 4 Oe, in good agreement with data in the literature.^86^ Low *H*_c_ and σ_r_ (3.5 ± 0.2 emu/g) and low hysteresis losses are characteristic of soft magnetic materials, whose typical representative is Fe_3_O_4_. *H*_c_ is linked to the small size of magnetite crystallites. The finding that the particle consists of agglomerates of magnetite crystallites is clearly illustrated in Figure 3B. The size of the crystallites of magnetite utilized in this study does not exceed 36.1 nm according to our previous publication.^84^ The greatest coercive force can be attained when the size of magnetite crystallites is equal to one domain.^86^

**Table 4:** Magnetic Properties of the Fabricated Composites

sample	σ_s_, emu/g	σ_r_, emu/g	*H*_c_, Oe
Fe_3_O_4_–rGO	96.27 ± 1.42	3.50 ± 0.20	60 ± 4.00
PHB/Fe_3_O_4_–rGO_G27_	6.50 ± 0.39	0.50 ± 0.03	160 ± 9.60
PHB/Fe_3_O_4_–rGO_G21_	6.83 ± 0.41	0.46 ± 0.03	113 ± 6.78

Saturation magnetization of the PHB/Fe_3_O_4_–rGO polymer scaffolds (Figure 10B) turned out to be 6.50 ± 0.39 and 6.83 ± 0.41 emu/g for the 27G and 21G needles, respectively. Low σ_s_ in polymer scaffolds is explained by the low concentration of the Fe_3_O_4_–rGO phase (8 wt %). A low Fe_3_O_4_–rGO content in matrices weakens the interaction between magnetite particles and thereby also possibly decreasing σ_s_.^87^ Remanent magnetization σ_r_ showed the same trend.

Coercive force *H*_c_ corresponding to the magnetic field, which is necessary to eliminate remanent magnetization, significantly increased: from 60 ± 4 Oe for the Fe_3_O_4_–rGO composite to 160 ± 10 and 113 ± 7 Oe for the scaffolds obtained with the 27G and 21G needles, respectively. It is known that the coercive force significantly depends on the size and shape anisotropy of crystallites of magnetite^88^ as well as on the presence of impurities in the material.^86^ The highest *H*_c_ is reached in the single-domain state of the material. For Fe_3_O_4_, the size of one domain is ≈128 nm.^88^ A further decrease in the crystallite size causes a sharp drop of *H*_c_ to zero.^89^ The size of magnetite crystallites in the composite analyzed in this work is ∼36 nm.^84^ Nonetheless, the coercive force is 60 Oe because crystallites do not have an ideal spherical shape, and the deviation from sphericity for single-domain particles affects the coercive force.^88^ The presence of rGO in composites also raises *H*_c_. An increase in the coercive force for PHB/Fe_3_O_4_–rGO composites is related to the rupture of chain aggregates of particles in the composite, which cease to interact magnetostatically with each other, with a decrease in the degree of filling, which effectively enhances the magnetic anisotropy field and raises *H*_c_. An increase in *H*_c_ indicates that the PHB fiber resists the equalization of the filler’s magnetic moment. Consequently, composites with a lower filler content hardly demagnetize in contrast to composites with a higher filler content.^90^

In a comparison of the scaffolds with different fiber diameters, no significant differences in σ_s_ and σ_r_ were detected. Nonetheless, a considerable increase in *H*_c_ from 113 ± 6.78 to 160 ± 9.60 Oe was documented when the fiber diameter diminished from 3.0 ± 0.5 to 1.7 ± 0.3 μm, respectively, for the composite scaffolds. A decrease in the fiber diameter and the diameter of the needle promotes breaks in the chain aggregates of particles in the composite, thereby inducing an even greater increase in the anisotropy fields and hence in *H*_c_. This finding supports our supposition that the coercive force depends on the presence of Fe_3_O_4_–rGO in the materials.

The XPS analysis was performed to evaluate alterations in the surface composition of PHB scaffolds under the influence of the changes in the fiber diameter and/or the addition of magnetite particles and rGO. There is no difference in the elemental composition among all the scaffolds (Figure S2, survey XPS spectra). Notably, no iron was detectable on the surface of the composite scaffolds. Taking into account that XPS-sensitive depth varies by up to 10 nm in a polymer,^33^ this result means the absence of magnetite particles on the fiber surface.

**Figure 11:**
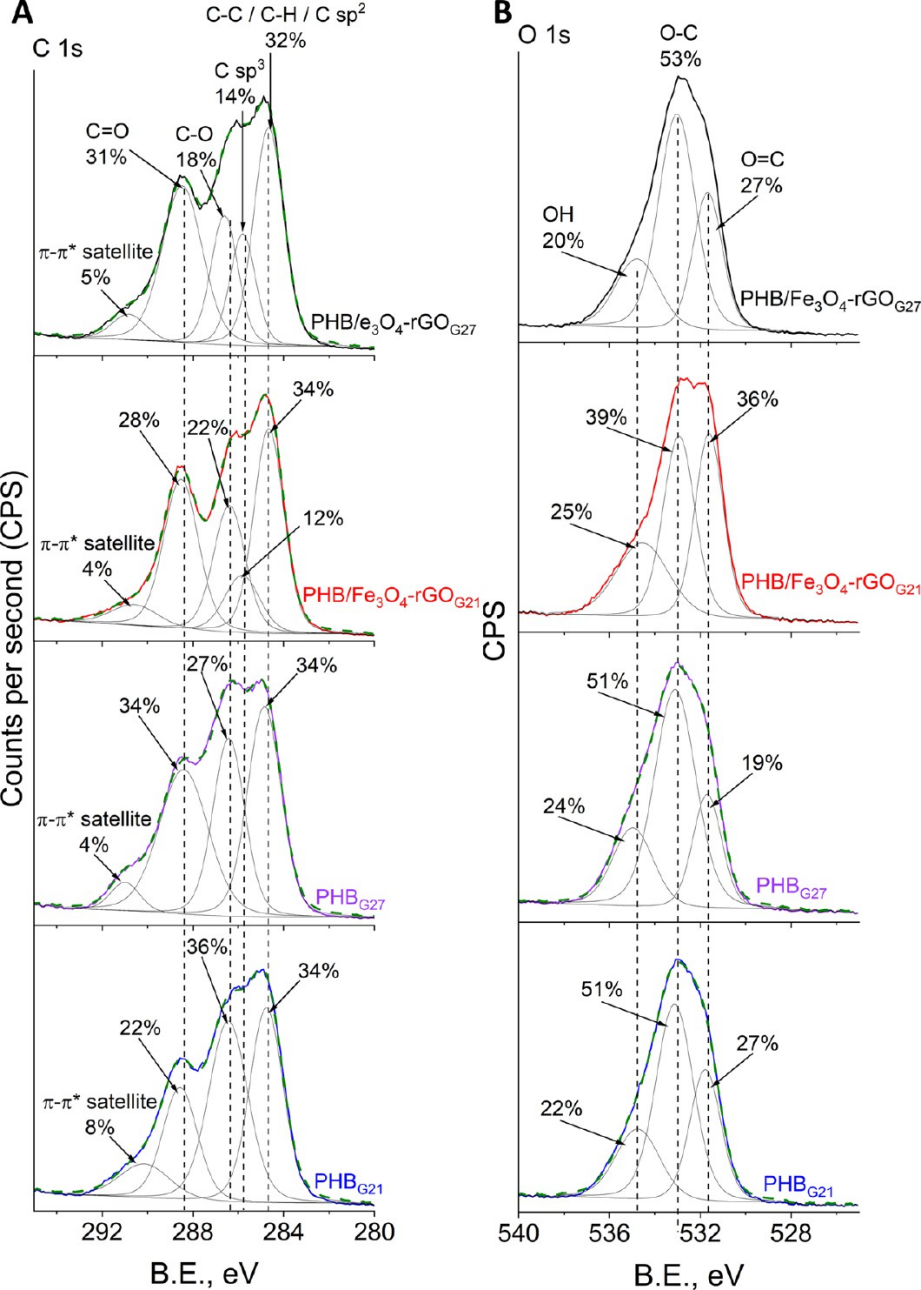
High-resolution XPS spectra of C 1s (A) and O 1s (B) regions for pristine and magnetic composite PHB scaffolds formed via 21G and 27G needles.

High-resolution XPS spectra of C 1s and O 1s regions for pure and composite scaffolds with different fiber diameters are presented in Figure 11. Independently from the fiber diameter, the fitting of the C 1s region for pure scaffolds yielded all typical peaks corresponding to PHB as follows (Figure 11A): C–C–C/C–H (285 eV), C–O (286.6 eV), and C=O (288.8 eV).^91^ Our analysis of the O 1s region confirmed the typical oxygen functional groups, such as C–O (533 eV) and C=O (531.7 eV).^91^ The addition of magnetite particles and rGO altered the C 1s shape profile, for example, by increasing the peak over 285 eV. This finding can be explained by the contribution of C sp^2^ (285 eV) and C sp^3^ from rGO.^66^ A greater contribution of the polar C=O functional group to the O 1s region was revealed for composite scaffolds and can also be attributed to rGO according to the literature.^14^ It is worth mentioning that the presence of all these functional groups on the surface of rGO, which was used in the present study, is demonstrated in the Supporting Information (Figure S2). These changes in the C 1s and O 1s regions of the composite scaffolds may indicate that rGO flakes tend to be located near the fiber surface. It is reported that electrostatic attraction of rGO flakes to the surface of electrospun fibers may be expected upon electrospinning when a high electric field is applied.^14^ It is noteworthy that these alterations in C 1s and O 1s regions were more pronounced in the composite fibers with the greater diameter (PHB/Fe_3_O_4_–rGO_G21_), and this phenomenon can be explained by a larger surface area as compared to the scaffolds with the smaller fiber diameter (PHB/Fe_3_O_4_–rGO_G27_).

**Figure 12:**
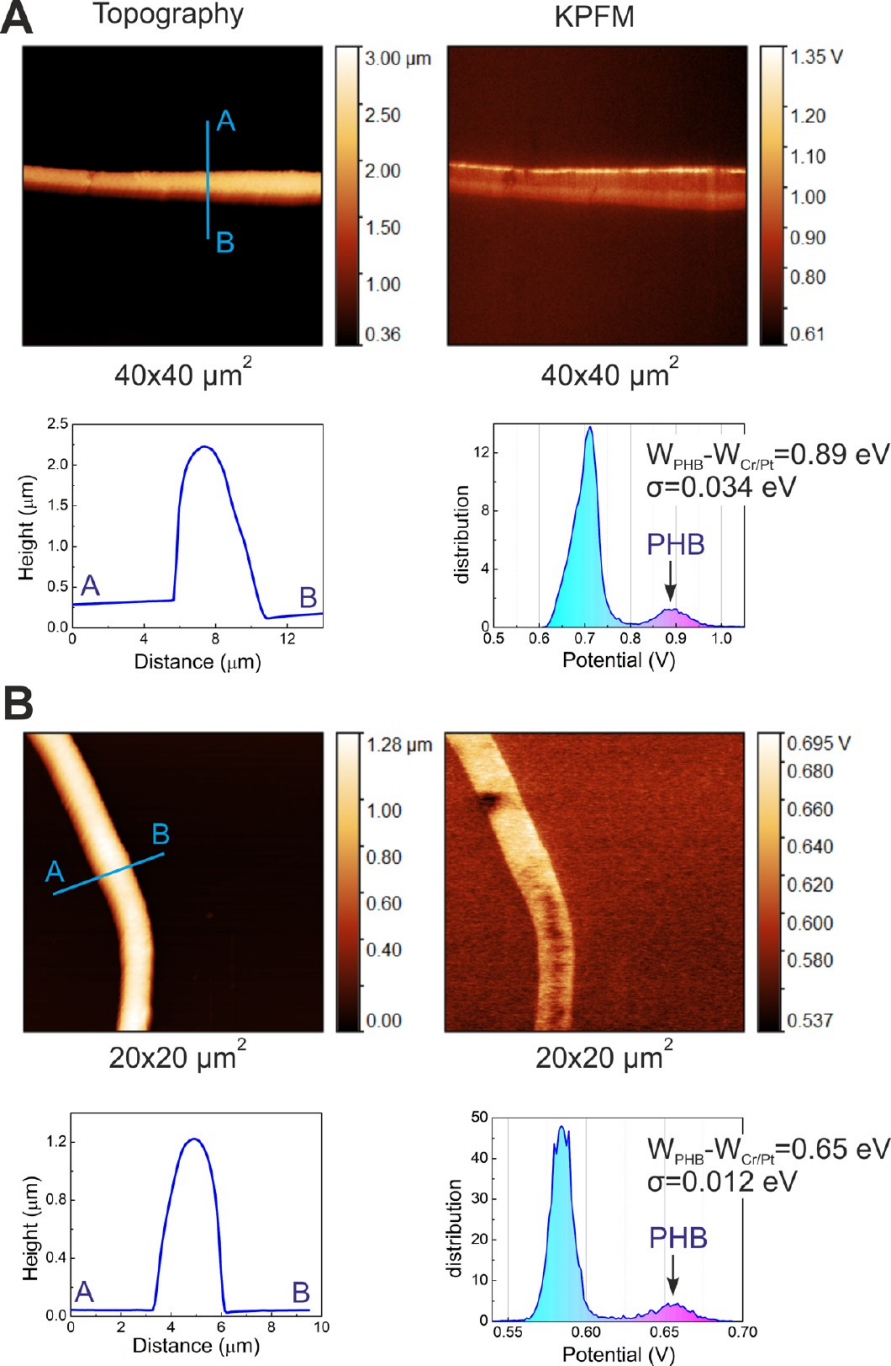
Topography and KPFM images of PHB/Fe_3_O_4_–rGO_G21_ (A) and PHB/Fe_3_O_4_–rGO_G27_ fibers (B).

Figure 12 reveals the results of the surface topography (AFM) and electric-potential distribution (KPFM) analyses for the hybrid fibers possessing different diameters. According to the topography profiles, homogeneous ellipsoid microfibers are present in both the composites PHB/Fe_3_O_4_–rGO_G21_ and PHB/Fe_3_O_4_–rGO_G27_ fibers. Nevertheless, an almost twofold difference in height was noticed between composite fibers fabricated using needles of different sizes, confirming the results of the SEM analysis (Figure 5). Qualitatively, there is no sufficient difference in the distribution of surface electric potential between the two types of fibers (21G and 27G). Quantitatively, the average surface electric potential significantly declined (from 0.89 ± 0.034 to 0.65 ± 0.012 eV) with the diminishing fiber diameter of composites.

The author of ref (13) reports declining surface electric potential of pure PLLA fibers with their diminishing diameter, which was explained by the declining crystallinity of the scaffolds with decreasing fiber diameter, whereas the crystalline polymer state had a more regular dipole order than the amorphous one did. Indeed, the reduction in the fiber diameter in the case of the PHB polymer also diminishes the degree of crystallinity, as shown in Table 3. Despite the significant difference in the diameter of fibers, the two composite PHB/Fe_3_O_4_–rGO_G21_ and PHB/Fe_3_O_4_–rGO_G27_ scaffolds have similar degrees of crystallinity (Table 3), and the reason is the addition of magnetite and rGO. A significantly higher surface electric potential was recently demonstrated in hybrid PHB scaffolds after the introduction of rGO flakes, resulting in a greater number of polar C=O functional groups at the surface of the fibers.^92^ In the present work, a similar increase in the number of polar C=O functional groups at the surface of fibers was seen after the doping of the scaffolds with rGO (Figure 11).

**Figure 13:**
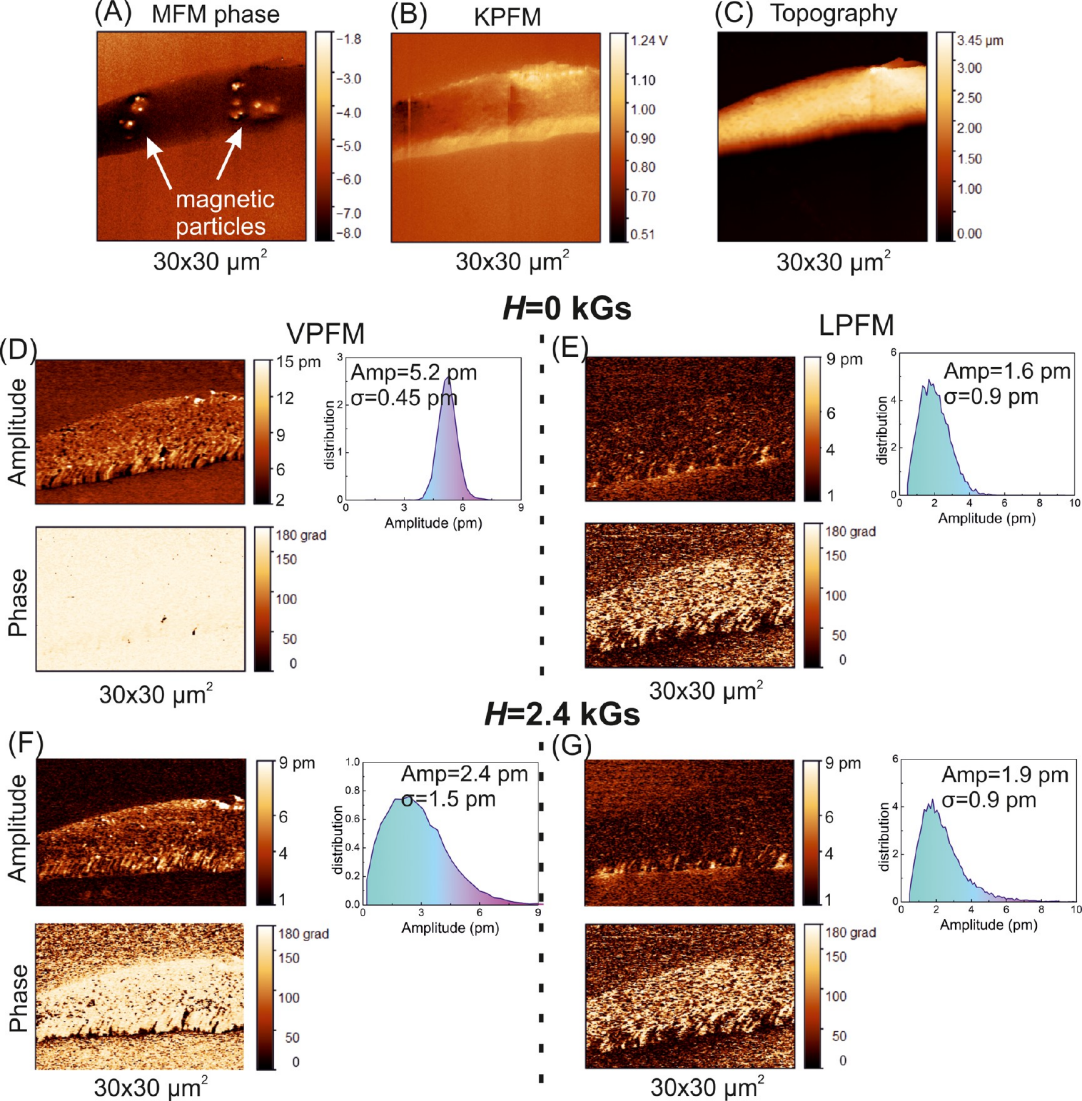
MFM phase (A), KPFM (B), and topography (C) images of PHB/Fe_3_O_4_–rGO_G21_ fibers. VPFM (D,F) and LPFM (E,G) images of the amplitude and phase of PHB/Fe_3_O_4_–rGO_G21_ fibers without (*H* = 0 kGs) and with a magnetic field (*H* = 2.4 kGs). Bias voltage U_ac_ = 24 V.

**Figure 14:**
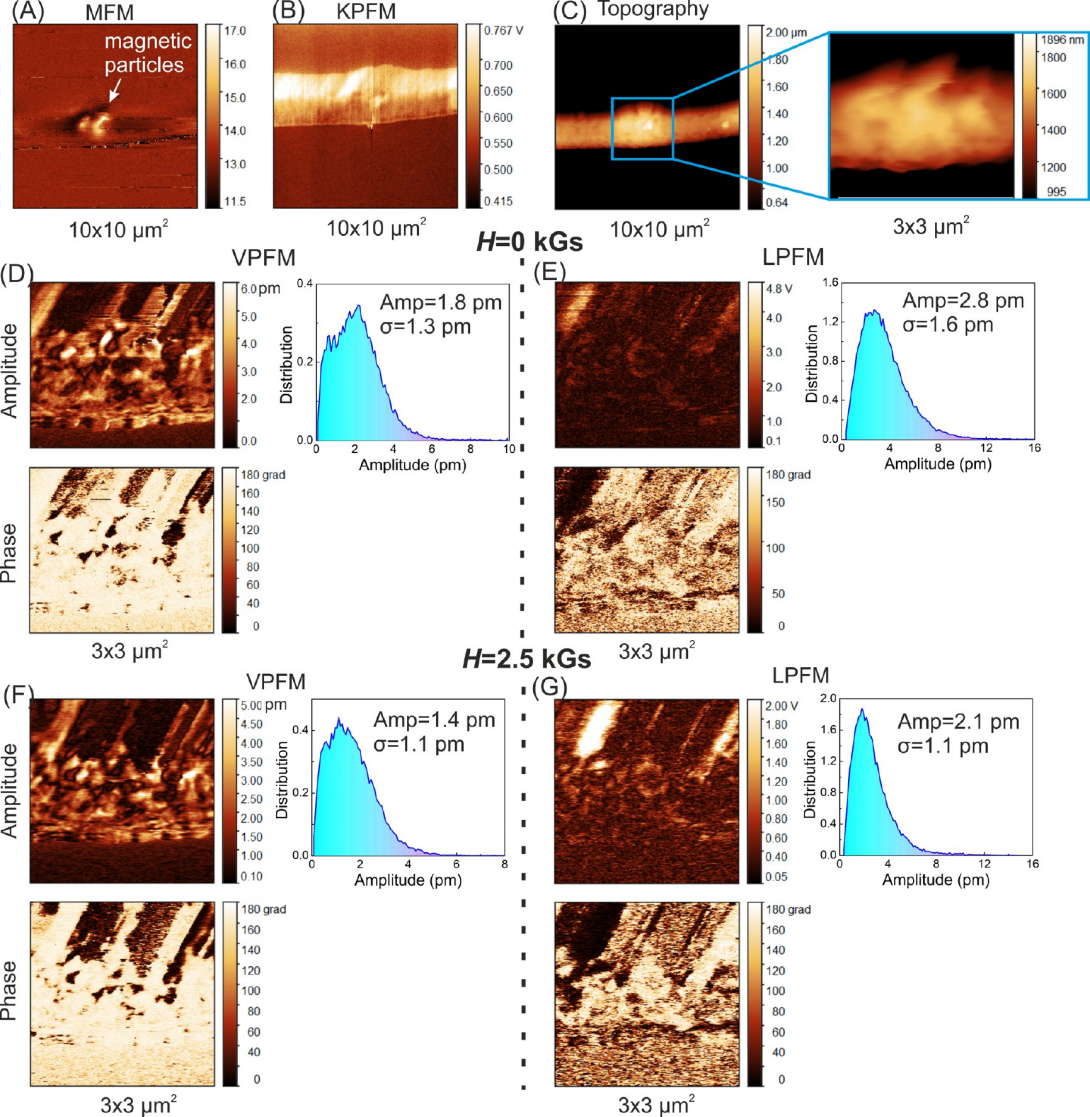
MFM phase (A), KPFM (B), and topography (C) images of PHB/Fe_3_O_4_–rGO_G27_ fibers. VPFM (D,F) and LPFM (E,G) images of the amplitude and phase of PHB/Fe_3_O_4_–rGO_G27_ fibers without (*H* = 0 kGs) (D,E) and with a magnetic field (*H* = 2.5 kGs) (F,G), including an amplitude distribution. Bias voltage U_ac_ = 24 V.

Figures 13a and 14a show typical phase MFM images, which confirm the presence of magnetite particles inside the polymer fibers. At the same time, according to the observed location of the magnetic particles in the fibers, there is no noticeable impact of the magnetic particles on the surface potential (Figures 13b and 14b) and topography (Figures 13c and 14c) of the fibers, for example, charge accumulation or bumps, respectively. Even though the magnetic particles are located close to the fiber surface, the particles are likely covered with a polymer layer.

Figures 13d,e and 14d,e present PFM images of the amplitude and phase for the vertical and lateral signals from hybrid fibers without and with the application of the external magnetic field. The presence of contrast variations in the PFM images is explained by a polydomain state in the hybrid PHB fibers. Typical contrast (a polydomain state) in vertical PFM (VPFM) and lateral PFM (LPFM) of polymers, such as PHB and PVDF, has been reported too.^92−94^ Our analysis of the amplitude distribution in both VPFM and LPFM data from fibers with different diameters suggested that the effective VPFM response is higher for the larger diameter (21G) than for the smaller diameter (27G). The mean effective VPFM response ranged from 0.12 to 0.22 pm/V for group 21G and from 0.05 to 0.1 pm/V for group 27G. By contrast, for the LPFM signal, we documented the opposite tendency, the mean effective value was less for the larger diameter (21G: 0.06–0.08 pm/V) and greater for the smaller diameter (27G: 0.067–0.13 pm/V). We ascribed this effect to the electrostatic contribution to the LPFM signal from sloping parts of the fibers. The influence of the magnetic field on the electromechanical activity of the fibers was below the sensitivity of our PFM system: the observed differences in VPFM and LPFM signals with and without the magnetic field were within the instrumental error.

An in-plane piezoresponse in hybrid magnetic PHB scaffolds is expected due to the observed α-phase with orthorhombic crystalline symmetry corresponding to space group *P*2_1_2_1_2_1_, which possesses shear piezoelectric components (*d*_14_, *d*_25_, *d*_36_).^95^ In turn, the presence of an out-of-plane piezoresponse of the fabricated fibers can be explained by a contribution of the following possible mechanisms: (i) existence of the zigzag confirmation with a hexagonal unit cell of the *P*3_2_1 space group possessing a variety of piezoelectric components, including shear (*d*_14_, *d*_25_, *d*_26_), (*d*_12_), and normal (*d*_11_) tensors;^95^ (ii) a specific contribution of dipoles from interactions between polymer chains and rGO;^96^ and (iii) randomly oriented lamellae and the bucking effect from the lamellae oriented along the surface.^97^

Our examination of the effective local in-plane piezoresponses for the hybrid magnetic PHB fibers with different diameters pointed to the absence of a sufficient difference (Table 5). Additionally, there was no impact of the external DC magnetic field with a strength of up to 2.5 kGs on either in-plane or out-of-plane piezoresponses of both types of hybrid fibers, that is, PHB/Fe_3_O_4_–rGO_G21_ and PHB/Fe_3_O_4_–rGO_G27_. On the other hand, a decline in a local piezoresponse from 205.8 ± 11.9 to 87.1 ± 3.3 pm in a DC external magnetic field that is varied from 0 to 2000 Oe, respectively, has been reported for nonbiodegradable PVDF fibers with magnetite particles.^98^ In the current study, however, a much lower amount of magnetic particles (6 wt % of magnetic particles with rGO) was introduced as compared to the literature data, where investigators added 10 wt % of magnetic particles.^98^

**Table 5:** Effective Local Piezoresponses for Scaffolds with Different Fiber Diameters with and without Exposure to the Magnetic Field

		piezoresponse	
magnetic field strength (kGs)	scaffolds	VPFM (pm/V)	LPFM (pm/V)
0	PHB/Fe_3_O_4_–rGO_G21_	0.12–0.22	0.06–0.08
	PHB/Fe_3_O_4_–rGO_G27_	0.05–0.10	0.07–0.13
2.4	PHB/Fe_3_O_4_–rGO_G21_	0.10–0.17	0.06–0.09
	PHB/Fe_3_O_4_–rGO_G27_	0.06–0.10	0.08–0.13

## 4 Conclusions

The effects of a hybrid magnetic Fe_3_O_4_–rGO filler and of the fiber diameter on structural, mechanical, magnetic, and piezoelectric properties of PHB scaffolds were revealed. A Fe_3_O_4_–rGO composite with high saturation magnetization, 96.27 ± 1.42 emu/g, was synthesized by the in situ coprecipitation method, which amplified the defect density of rGO. Defect-free pure and composite PHB scaffolds were successfully fabricated via electrospinning with fiber diameters of 1.6 ± 0.3 and 2.4 ± 0.5 μm for pure PHB scaffolds and 1.7 ± 0.3 and 3.0 ± 0.5 μm for composite PHB/Fe_3_O_4_–rGO scaffolds, by means of 27G and 21G needles, respectively. After characterization of the fabricated Fe_3_O_4_–rGO filler and electrospun scaffolds, the following conclusions were drawn:

- A decrease in the needle diameter (from 0.51 to 0.2 mm) and the addition of the Fe_3_O_4_–rGO filler lowered the crystallinity of the scaffolds. At the same time, the Fe_3_O_4_–rGO filler reduced the (020) and (110) crystallite size of the orthorhombic α-phase of PHB scaffolds.
- The decrease in the fiber diameter enhanced the ductility and strength of the electrospun scaffolds. Elongation at break improved from 10 ± 1.5 to 15 ± 3.0% for pure PHB scaffolds and from 7.8 ± 2.6 to 18.5 ± 5.7% for the PHB/Fe_3_O_4_–rGO composite scaffolds. The higher ductility of the finer fibers is attributable to the better ability of such fibers to absorb a considerable amount of energy before failure. The thinner fibers (obtained with the 27G needle) possess ultimate strengths of 2.50 ± 0.27 and 1.05 ± 0.18 MPa in the pure and composite scaffolds, respectively; these values are more than twice higher than those of the scaffolds fabricated via the 21G needle. Elongation at break slightly increased after the addition of Fe_3_O_4_–rGO composite fillers: from 15.0 ± 3.0 to 18.5 ± 5.7% for the scaffolds electrospun with the needles 0.2 mm in diameter. The addition of Fe_3_O_4_–rGO fillers diminished ultimate strength from 1.35 ± 0.10 to 0.49 ± 0.15 MPa and from 2.50 ± 0.27 to 1.05 ± 0.18 MPa for the 21G and 27G groups, respectively. Young’s moduli are lower too in the composite scaffolds compared to the pure ones in both the 21G and 27G groups.
- Surface electric potential of the magnetoactive PHB/Fe_3_O_4_–rGO composite scaffolds significantly increased from 0.650 ± 0.012 to 0.890 ± 0.034 V with the enlarged fiber diameter owing to a larger amount of polar functional surface groups. There was no influence of microfiber sizes on either out-of-plane or in-plane effective local piezoresponses for the hybrid magnetic PHB fibers. Meanwhile, the newly developed scaffolds have high saturation magnetization: 6.50 ± 0.39 and 6.83 ± 0.41 emu/g for the scaffolds electrospun with the 27G and 21G electrospinning needles, respectively.

Thus, the introduction of the magnetic Fe_3_O_4_–rGO composite filler into PHB scaffolds did not affect the piezoresponse of the scaffolds but imparted pronounced magnetic properties. Additionally, the ductility and surface electric potential of the magnetoactive electrospun PHB/Fe_3_O_4_–rGO scaffolds can be controlled by varying the fiber diameter. Therefore, the proposed magnetic PHB/Fe_3_O_4_–rGO scaffolds, which can provide external mechanical and electrical stimuli, are promising candidates for bone tissue engineering.

## Supporting Information Available

Raman spectrum of rGO and XPS survey spectra for pure PHB and composite PHB/Fe_3_O_4_–rGO scaffolds formed with G21 and G27 electrospinning needles (PDF)

## Notes

The raw/processed data required to reproduce these findings cannot be shared at this time as the data also forms part of an ongoing study.
