# Supplementary material for: Comprehensive Study on the Reinforcement of Electrospun PHB Scaffolds with Composite Magnetic Fe3O4–rGO Fillers: Structure, Physico-Mechanical Properties, and Piezoelectric Response
Source: ACS Omega. 2022 Nov 4;7(45):41392–411. doi: 10.1021/acsomega.2c05184 (PMC9670262; doi:10.1021/acsomega.2c05184)
Supplement: Supplementary file 1 — ao2c05184_si_001.pdf [file ao2c05184_si_001.pdf]

Supporting information

A comprehensive study on the reinforcement of electrospun PHB scaffolds with composite magnetic Fe_3O_4-rGO filler: structure, physico-mechanical properties, and piezoelectric response

Artyom S. Pryadko[1], Yulia R. Mukhortova[1], Roman V. Chernozem[1], Lada E. Shlapakova[1], Dmitry V. Wagner[2], Konstantin Romanyuk[4,6], Evgeny Y. Gerasimov[5], Andrei Kholkin[3,6], Roman A. Surmenev[1,6,*], and Maria A. Surmeneva[1,6,*]

[1] Physical Materials Science and Composite Materials Center, Research School of Chemistry & Applied Biomedical Sciences, Tomsk Polytechnic University, Tomsk 634050, Russia

[2] Tomsk State University, Tomsk 634050, Russia

[3] School of Natural Sciences and Mathematics, Ural Federal University, Ekaterinburg 620000, Russia

[4] Department of Physics & CICECO−Aveiro Institute of Materials, University of Aveiro, Aveiro 3810-193, Portugal

[5] Boreskov Institute of Catalysis SB RAS, Novosibirsk 630090, Russia

[6] International Research & Development Center of Piezo- and Magnetoelectric Materials, Research School of Chemistry and Applied Biomedical Sciences, Tomsk Polytechnic University, Tomsk 634050, Russia

*corresponding authors: surmenevamaria@mail.ru (Maria A. Surmeneva); rsurmenev@mail.ru (Roman A. Surmenev)

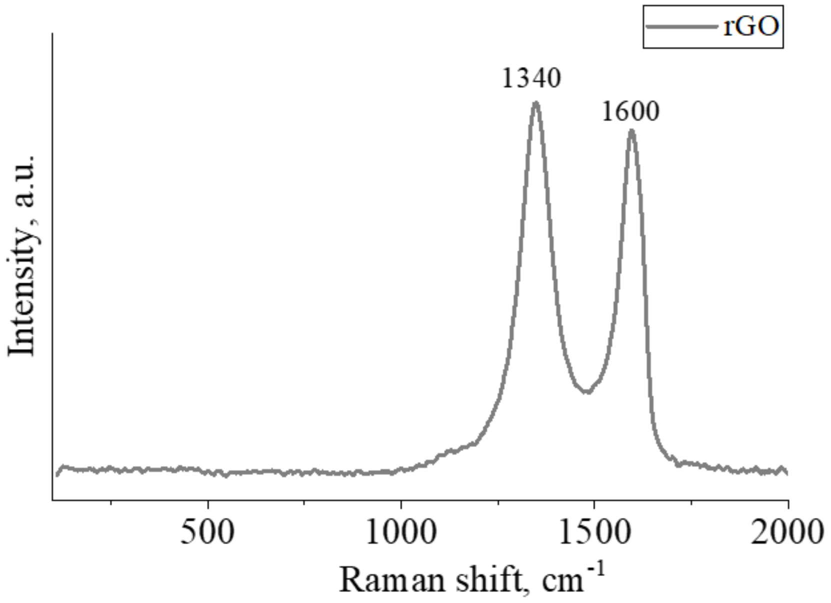

Figure S1. A typical Raman spectrum of rGO

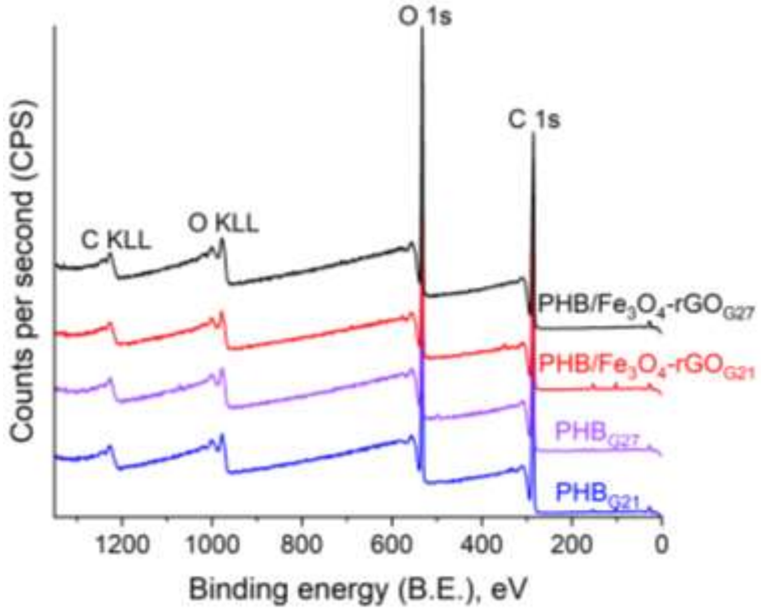

Figure S2. XPS survey spectra for pure PHB and composite PHB/Fe_3O_4-rGO scaffolds formed with G21 and G27 electrospinning needles
